# Circular *GOLPH3* RNA exerts oncogenic effects *in vitro* by regulating the miRNA-1299/LIF axis in oral squamous cell carcinoma

**DOI:** 10.1080/21655979.2022.2067288

**Published:** 2022-04-28

**Authors:** Chen Zou, Xia Li, Haigang Wei, Siyuan Wu, Jing Song, Zhe Tang, Hailing Luo, Xiaozhi Lv, Yilong Ai

**Affiliations:** a School of Medicine, Foshan Stomatological Hospital, Foshan University, Foshan, Guangdong, China; b Department of Oral and Maxillofacial Surgery, NanFang Hospital, Southern Medical University, Guangzhou, Guangdong, China

**Keywords:** CircGOLPH3, miR-1299, LIF, oncogenic properties, oral squamous cell carcinoma

## Abstract

Circular RNAs, which are a novel subclass of noncoding RNAs, are reported to be involved in various biological processes. Aberrant expression of circular RNAs may promote cancer progression. The function of circular *GOLPH3* RNA (circGOLPH3) in oral squamous cell carcinoma (OSCC) is unclear. In this study, the circGOLPH3 levels in OSCC cell lines were determined using quantitative real-time polymerase chain reaction (qRT-PCR). Gain-of-function and loss-of-function experiments were performed to evaluate the roles of circGOLPH3 in OSCC. Cell counting kit 8, migration, and invasion assays were performed to determine the functions of circGOLPH3. The mechanism of circGOLPH3 in OSCC was investigated using qRT-PCR, western blotting, luciferase activity, and RNA pull-down analyses. Furthermore, the function of circGOLPH3 *in vivo* was evaluated. circGOLPH3 derived from *GOLPH3* was mainly localized to the cytoplasm and exhibited high stability. The expression of circGOLPH3 was upregulated in OSCC cells. circGOLPH3 promoted the growth of OSCC *in vitro* and *in vivo*. Additionally, circGOLPH3 upregulated OSCC cell migration and invasion. Mechanistically, circGOLPH3 functioned as a microRNA sponge and downregulated miR-1299 expression. miR-1299 downregulated the expression of *LIF* by targeting its 3’-untranslated region. Inhibition of the circGOLPH3/miR-1299/LIF axis suppressed the growth, migration, and invasion of OSCC cells. These findings indicate that the circGOLPH3/miR-1299/LIF axis promotes OSCC cell growth, migration, and invasion and that this axis is a potential therapeutic target for OSCC.

## GRAPHICAL ABSTRACT

**Figure:**
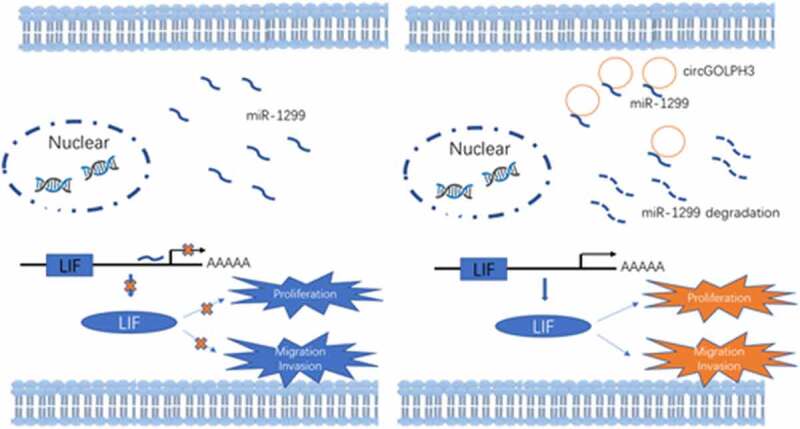

## Highlights

- circGOLPH3 expression is upregulated in oral squamous cell carcinoma (OSCC) cells.
- circGOLPH3 promotes OSCC cell proliferation, migration, and invasion.
- circGOLPH3/miR-1299/LIF axis exerts oncogenic effects in OSCC.

## Introduction

Oral cancer, a malignancy with a global caseload of more than 500,000, is the eighth most common cancer and the fifteenth most common cause of mortality among patients with cancer [1]. In particular, oral squamous cell carcinoma (OSCC) accounts for 90% of all oral cancer cases [2].

Recent developments in the diagnostic and therapeutic strategies for OSCC have not markedly decreased the mortality rates of OSCC. The 5-year survival rate of patients with OSCC is approximately 20% for late-stage disease owing to the high frequency of metastasis and recurrence [3,4]. The invasive epithelial cells contribute to the metastasis of OSCC [5]. The mechanisms underlying the tumorigenesis of OSCC have not been elucidated. Thus, there is a need to examine OSCC metastasis and recurrence.

Circular RNA (circRNA), a novel class of noncoding RNA [6], was considered a nonfunctional and aberrant product of RNA splicing in the 1970s [7,8]. Rapid development of high-throughput sequencing technologies has enabled the identification of several circRNAs in various tissues [9]. circRNAs have a stable closed-loop structure, which is formed by covalent bonds between the 3’ and 5’ ends [7]. The lack of 3’ termini protects circRNAs against exonuclease digestion. Thus, the stability of circRNAs is higher than that of linear mRNAs. circRNAs are rich in microRNA binding sites and may function as microRNA sponges to suppress the expression of microRNA [10]. Various studies have reported that circRNAs have critical functions in several physiological processes, including cell differentiation, proliferation, and survival [11]. Recent studies have reported the roles of circRNAs in pathological conditions, especially tumors. circRNA is reported to play important roles in breast [12], lung [13], gastric [14], and liver cancers [15]. Elucidation of the functions of circRNAs will provide useful insights into the complex molecular mechanisms underlying tumor progression. Limited studies have examined the function and role of circRNA in OSCC [16].

CircGOLPH3 (hsa_circ_0001470) is derived from *GOLPH3*, which plays an important role in the trafficking of the Golgi membrane and cargo transportation from the Golgi body to the plasma membrane [17–19]. The length of circGOLPH3, which is located in chr5:32135677–32143986, is 247 bp. Here, the function of circGOLPH3 in OSCC was examined. The expression levels of circGOLPH3 were examined in OSCC cell lines and oral epithelial keratinocyte cells. The roles of circGOLPH3 in OSCC progression, including migration, invasion, and immune response, were examined using gain-of-function and loss-of-function experiments. The findings of this study suggest that circGOLPH3 may play important roles in OSCC cell migration and modulate the immune microenvironment.

This study aimed to examine the expression and function of circGOLPH3 in OSCC. A panel of biological experiments was performed to determine the competing endogenous RNA network involved in circGOLPH3-mediated OSCC progression. We hypothesized that circGOLPH3 functions as a carcinogenic circRNA to regulate OSCC cell proliferation, migration, and invasion through the miR-1299/LIF axis. circRNAs are potential tumor markers and therapeutic targets owing to their structural stability. This study demonstrated that circGOLPH3 promotes the carcinogenic properties of OSCC cells *in vitro*. This is the first study to report the function of circGOLPH3 in OSCC. Thus, circGOLPH3 might provide new thoughts for for OSCC.

## Materials and methods

### Cell culture

The human OSCC cell lines HSC3, UM1, HN4, SCC9, SCC25, CAL27, SCC15, and normal oral epithelial keratinocyte (HOK) cells were obtained from the cell bank of the Chinese Academy of Sciences (Shanghai, China). All the cell lines were cultured under the instruction of ATCC. All cell lines were cultured at 37°C with 5% CO2 [20].

### Cell transfections

CircGOLPH3 targeting siRNA was ATTACTTAGTGGGTTACACAT. Control siRNA was ATGCTAACGAAGTCCAGTAAT. LIF siRNA was obtained from Shanghai Genechem Co.,Ltd. The overexpression plasmids of circGOLPH3 and LIF were produced by GenePharma (Suzhou, China) by using pcDNA3.1 plasmids. The indicated cells were transfected by using Lipofectamine 2000.

### Proliferation assay

Proliferation was measured through using Cell Counting Kit‑8 (CCK8) assay. 2 000 cells were plated into 96-well plates. 10 ul CCK8 was added per well, and the cells were incubated 2 hours under the instruction of the Kit. Absorbance values at 450 nm were detected by a microplate reader (Bio199 Rad). Experiment was repeated three times [21].

### Western blot

Total protein was collected by PIPA lysis buffer and 20ug protein was electrophoresed in SDS-PAGE gels (10%). The NC membranes were incubated with primary antibody for 12 hours at 4°C, and with the second antibody for 2 hours at 37°C. The primary antibodies used were: LIF (ab172023, abcam, Cambridge, MA, USA), GOLPH3 Polyclonal antibody (Proteintech Group, Chicago, IL, USA). The bands were measured by ECL western blot kit [22].

### RNA extraction and qRT-PCR

OSCC and normal cell RNA was extracted using TRIzol (Invitrogen, Carlsbad, CA, USA) according to manufacturer’s instruction. ND-2000 (NanoDrop, Wilmington, USA) was used to determine the RNA quality and concentration. We performed the cDNA synthesis with random primers and Oligo(dT) using the PrimeScript RT Reagent Kit following the protocol of the manufactures’ instructions (Takara Bio, Nojihigashi, Kusatsu, Japan). qRT-PCR was applied detect relative RNA levels by SYBR Kit (Roche). All expressions were normalized to internal β-actin. The results were analyzed by ΔΔCt method [23].

The primers are as following:

**Table:** 

Gene	Primer
CircGOLPH3 Primer 1	5’TCCAACAGGGGATGTTCTTC3’
CircGOLPH3 Primer 2	5’ATCATCTGGATTACGTGGCTG 3’
Linear GOLPH3 Primer 1	5’GTTTCCTCATGACTGCCCCC 3’
Linear GOLPH3 Primer 2	5’CTTTCTTGATGAGGCGCTGC3’
LIF Primer 1	GTCTTGGCGGCAGTACACAG
LIF Primer 2	CCCACATCTGGACCCAACTC
MiR-145 Primer 1	GGTCGTATGCAAAGCAGGGTCCGAGGTATCCA
MiR-145 Primer 2	TCGTCCAGTTTTCCCAGG
MiR-1299 Primer 1	GCCGAG AGGGAGUGUGUCUUAAGGUCUU
MiR-1299 Primer 2	CTCAACTGGTGTCGTGGA
MiR-331 Primer 1	GCCGAG AAGAUCCUAUCCGGGUCCCCG
MiR-331 Primer 2	CTCAACTGGTGTCGTGGA
MiR-516 Primer 1	GCCGAG CUUUCACGAAGAAAGGAGCUCUU
MiR-516 Primer 2	CTCAACTGGTGTCGTGGA
MiR-569 Primer 1	GCCGAGUGAAAGGUCCUAAGUAAUUGA
MiR-569 Primer 2	CTCAACTGGTGTCGTGGA
MiR-578 Primer 1	GCCGAG UGUUAGGAUCUCGUGUUCUUC
MiR-578 Primer 2	CTCAACTGGTGTCGTGGA
MiR-600 Primer 1	GCCGAG CUCGUUCCGAGAACAGACAUUCA
MiR-600 Primer 2	CTCAACTGGTGTCGTGGA
MiR-633 Primer 1	GCCGAG AAAUAACACCAUCUAUGAUAAUC
MiR-633 Primer 2	CTCAACTGGTGTCGTGGA

### Migration and invasion assays

Migration assay was performed with uncoated Transwell chambers (Corning Life Sciences, Corning, NY).200000 cells in 100 μl serum-free DMEM were added to the upper chamber. The lower chamber was filled with 800 μl DMEM supplementary with 10% FBS. The transwell chambers were cultured for 48 hours. The cells were fixed with 4% PFA and stained with 0.5% crystal violet. Invasion assays were carried out as described above with chambers which were pre-coated with Matrigel matrix (Sigma-Aldrich, USA). Images were obtained by a microscope and analyzed by Image J [24].

### RNase R assay

We extracted the RNAs and treated the RNA with RNase R (20 U/ml, Epicenter) for 2 hours. Then the RNAs were detected by qRT-PCR [25].

### Luciferase assay

circGOLPH3 or LIF 3’UTR was cloned into pGL3 plasmid (Promega Corporation, Madison, WI, USA). QuickChange Mutagenesis Kit (Stratagene, La Jolla, CA, USA) was applied to synthesize the mutations of miR-1299 targeting sites in circGOLPH3 or LIF 3’UTR. All plasmids were co-transfected with mimics or inhibitor of miR-1299. Luciferase activities were measured by Luciferase Reporter Assay Kit (Promega) 48 hours after transfections. Renilla luciferase activity was also determined subsequently [26].

### RNA Pull-down

RNA pull-down assays were performed with biotin labeled control or circGOLPH3 using RNA Pull down Kit (Thermo Fisher Scientific). RNA pull-down assays were also carried out with biotin labeled LIF 3’ UTR.

### Fluorescence in situ hybridization

CircGOLPH3 expression in OSCC cells was measured using biotin-labeled probes. Hybridization was carried out with circGOLPH3 probes overnight [27]. Cells were analyzed using Zeiss fluorescence microscope.

### Actinomycin D assay

The cells were pre-treated with 0.2uM Act D (Sigma). The linear RNA and circular RNA levels were detected by qRT-PCR at 0 h, 4 h, 8 h,12 h,24 h [28].

### Subcellular fractionation location assay

Nuclear Extraction Kit (beyotime) was used to isolate nuclear and cytoplasmic RNA in OSCC cells following the manufactures’ introduction.

### Tumor xenografts experiments

6 -week-old nude mice were injected with 2.8 × 10^6^ NC and si-circGOLPH3 UM1 cells. Each group has six mice. Tumor growth rates were calculated by volume: V = length × width^2^/2. The length and width were calculated every 3 days. We recorded the tumor size for 30 days and then collected the tumors for photograph. All the experiments were approved by Experimental Animal Ethics Committee of Foshan Stomatological Hospital.

### Statistical analysis

All data are shown as mean ± SD. GraphPad Prism 6 (GraphPad Software Inc.) was used for statistical analysis. The correlations between circGOLPH3, miR-1299, and LIF expressions were analyzed by Spearman’s correlation coefficient. Comparison between two groups was analyzed by unpaired two-tailed Student’s t-test. Comparison between multiple groups was analyzed by ANOVA. p < 0.05 is identified as statistically significant [29,30].

## Results

We examined the expression of circGOLPH3 in OSCC cells. Gain-of-function and loss-of-function experiments revealed that circGOLPH3 promotes the proliferation, migration, and invasion of OSCC cells. circGOLPH3 exerts oncogenic effects through miR-1299/LIF. These results suggest that circGOLPH3 is a potential therapeutic target for OSCC.

### circGOLPH3 is upregulated in OSCC

**Figure 1.**
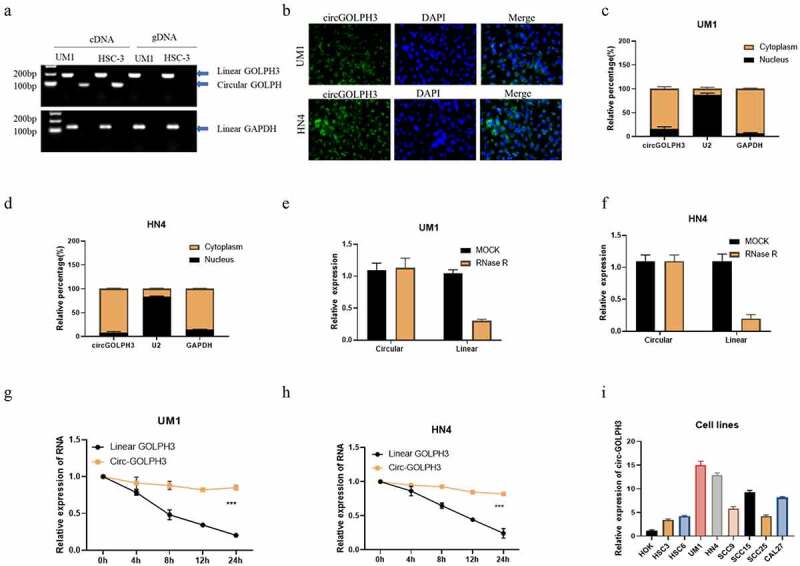
Expression of circular *GOLPH3* RNA (circGOLPH3) in oral squamous cell carcinoma (OSCC). (a) Specific primers were used to amplify circGOLPH3 and *GOLPH3* mRNA from complementary DNA and genomic DNA. (b) The cellular localization of circGOLPH3 in UM1 (up) and HN4 (down) cells was determined using RNA fluorescence *in situ* hybridization. (c–d) Cells were subjected to fractionation to isolate the nuclear and cytoplasmic fractions. circGOLPH3 levels in the nuclear and cytoplasmic fractions were determined using quantitative real-time polymerase chain reaction (qRT-PCR). (e–f) Total RNAs isolated from UM1 or HN4 cells were treated with MOCK or RNase R. The relative levels of circGOLPH3 and *GOLPH3* mRNA in the treated cells were determined using qRT-PCR. (g–h) Cells were treated with actinomycin D for different durations. The relative levels of circGOLPH3 and *GOLPH3* mRNA in actinomycin D-treated cells were determined using qRT-PCR. (i) The levels of circGOLPH3 in OSCC cells and human oral keratinocyte cells were determined.

The expression levels of circGOLPH3 in OSCC cell lines were examined. Divergent and convergent primers were used to amplify circGOLPH3 and *GOLPH3* mRNA. Linear *GOLPH3* RNA could be amplified from both genomic DNA and complementary DNA (cDNA) in UM1 and HSC-3 OSCC cell lines, whereas circGOLPH3 was amplified from only cDNA (Figure 1(a)). Fluorescence *in situ* hybridization analysis revealed that circGOLPH3 was mainly localized to the cytoplasm (Figure 1(b)). The expression levels of circGOLPH3 in the nuclear and cytoplasmic fractions of UM1 (Figure 1(c)) and HN4 (Figure 1(d)) cells were examined using quantitative real-time polymerase chain reaction (qRT-PCR). The stability of circGOLPH3 was examined using the RNase R and half-life assays (Figure 1(e-h)). circGOLPH3 was resistant to RNase R-mediated digestion (Figure 1(e,f)). Additionally, the half-life of circGOLPH3 was higher than that of linear *GOLPH3* RNA (Figure 1(g,h)). These findings indicated the high stability of circGOLPH3. circGOLPH3 levels in OSCC cells were significantly upregulated when compared with those in non-cancerous cells (Figure 1(i)). These findings indicate that circGOLPH3 is mainly localized to the cytoplasm and that it exhibits higher stability than linear RNA.

### circGOLPH3 exerts oncogenic effects in OSCC without influencing GOLPH3 protein level

**Figure 2.**
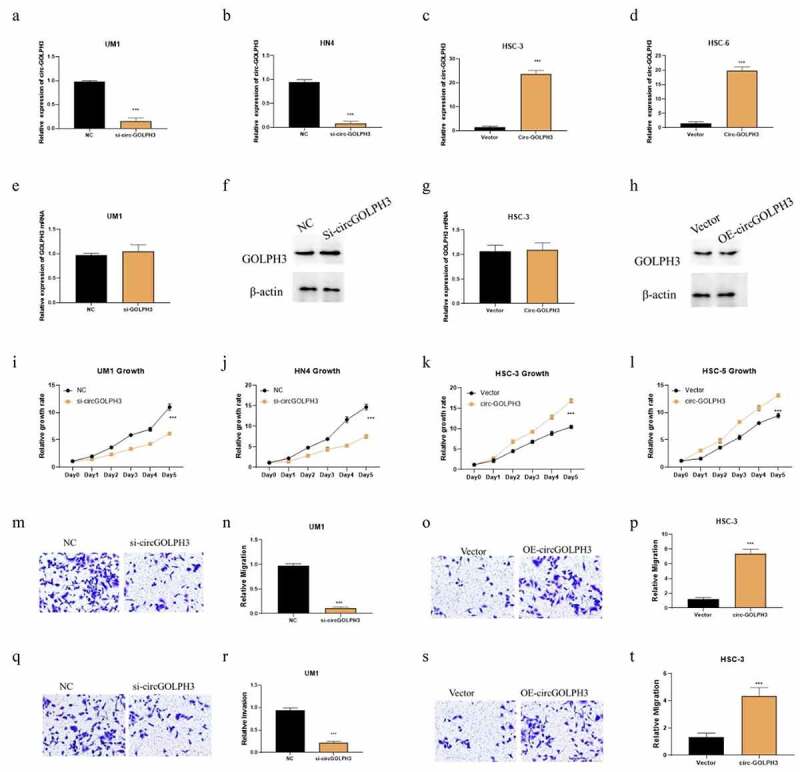
Circular *GOLPH3* RNA (circGOLPH3) promotes the proliferation, migration, and invasion of oral squamous cell carcinoma (OSCC) cells. (a–b) Short-interfering RNAs (siRNAs) targeting circGOLPH3 were transfected into UM1 or HN4 cells. The knockdown efficiency of siRNAs was determined using quantitative real-time polymerase chain reaction (qRT-PCR). (c–d) circGOLPH3 overexpression plasmids were transfected into HSC-3 or HSC-6 cells. The levels of circGOLPH3 in the circGOLPH3 overexpression plasmid-transfected cells were determined using qRT-PCR. mRNA and protein levels of GOLPH3 in UM1 (e–f) and HSC-3 cells (g–h). (i–l) Proliferation rates of the UM1 (i), HN4 (j), HSC-3 (k), and HSC-6 (l) cells were determined using the cell counting kit 8 assay. The effects of circGOLPH3 knockdown on UM1 cell migration (m–n) and the effects of circGOLPH3 overexpression on HSC-3 cell migration (o–p) were examined using the migration assay. UM1 (q–r) and HSC-3 (s–t) cells were subjected to the invasion assay.

Our results showed that UM1 and HN4 had higher expression level of circGOLPH3, while HSC-3 and HSC-6 had lower levels of circGOLPH3 (Figure 1(i)). Therefore, UM1 and HN4 cells were selected for loss-of-function experiments, while HSC-3 and HSC-6 cells were selected for gain-of-function experiments. The efficiency of circGOLPH3 knockdown in UM1 and HN4 cells was determined using qRT-PCR (Figure 2(a,b)). Transfection with circGOLPH3 overexpression plasmids upregulated circGOLPH3 levels in HSC-3 and HSC-6 cells (Figure 2(c,d)). Previous studies have reported that circRNA can regulate protein levels [31]. Hence, the effects of circGOLPH3 on GOLPH3 levels were examined using qRT-PCR and western blotting. Overexpression or knockdown of circGOLPH3 did not affect GOLPH3 levels (Figure 2(e-h)).

Next, cell counting kit 8 (CCK8), migration, and invasion assays were performed to examine the functional role of circGOLPH3 in OSCC. Overexpression of circGOLPH3 significantly promoted the proliferation of UM1 and HN4 cells (Figure 2(i,j)), whereas circGOLPH3 knockdown inhibited the proliferation of HSC-3 and HSC-6 cells (Figure 2(k,l)). Additionally, circGOLPH3 overexpression promoted the migration and invasion of UM1 cells (Figure 2(m,n,q,r)), whereas circGOLPH3 silence inhibited the migration and invasion of HSC-3 cells (Figure 2(o,p,s,t)).

### circGOLPH3 sponges miR-1299

**Figure 3.**
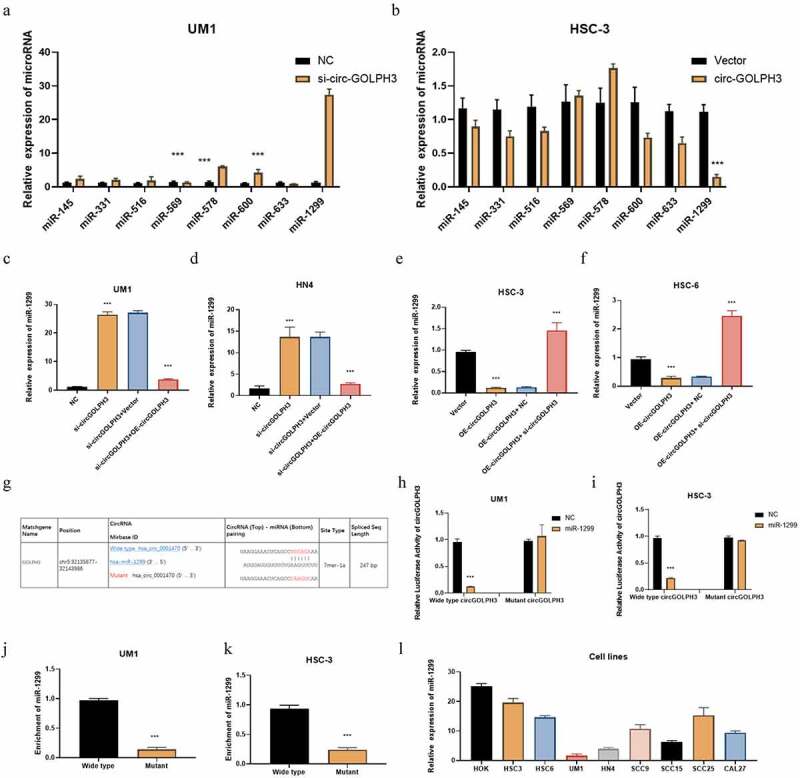
Circular *GOLPH3* RNA (circGOLPH3) functions as a sponge for microRNA-1299 (miR-1299). (a–b) The levels of the indicated microRNAs in UM1 and HSC-3 cells were determined using quantitative real-time polymerase chain reaction (qRT-PCR). (c–d) Empty vector or circGOLPH3 overexpression plasmid was transfected into si-circGOLPH3-transfected UM1 and HN4 cells. miR-1299 levels in the transfected cells were determined using qRT-PCR. (e–f) Short-interfering RNAs (siRNAs) targeting circGOLPH3 were transfected into circGOLPH3-overexpressing HSC-3 and HSC-6 cells. (g) The mutant binding sites of miR-1299 in circGOLPH3. (h–i) UM1 and HSC-3 cells were subjected to the luciferase assay to examine the effect of miR-1299 on the luciferase activities of circGOLPH3 with wild-type or mutant miR-1299-binding sites. (j–k) RNA pull-down assays were performed with circGOLPH3 harboring wild-type or mutant miR-1299-binding sites in UM1 and HSC-3 cells. (l) miR-1299 levels in oral squamous cell carcinoma cells were examined using qRT-PCR.

circRNAs are reported to sponge microRNAs and consequently regulate microRNA levels [32]. circGOLPH3 may play important roles in post-transcriptional regulation owing to its cytoplasmic localization. CircInteractome (https://circinteractome.nia.nih.gov) predicted that circGOLPH3 has eight potential target microRNAs. The levels of these eight possible target microRNAs in circGOLPH3-overexpressing and circGOLPH3 knockdown cells were determined using qRT-PCR. circGOLPH3 knockdown effectively upregulated the expression of miR-1299 (Figure 3(a)), whereas circGOLPH3 overexpression significantly downregulated the expression of miR-1299 (Figure 3(b)). To confirm whether circGOLPH3 specifically regulated miR-1299, rescue experiments were performed in UM1 and HN4 cells. circGOLPH3 overexpression mitigated the circGOLPH3 knockdown-induced upregulation of miR-1299 in UM1 and HN4 cells (Figure 3(c,d)). Meanwhile, circGOLPH3 knockdown mitigated the circGOLPH3 overexpression-induced downregulation of miR-1299 in HSC-3 and HSC-6 cells (Figure 3(e,f)).

The wild-type and mutated predicted binding sites for miR-1299 in circGOLPH3 are shown in Figure 3(g). The results of the luciferase assay revealed that miR-1299 downregulated the luciferase activity of wild-type circGOLPH3 but not that of mutant circGOLPH3 in UM1 and HSC-3 cells (Figure 3(h,i)). The results of the RNA pull-down assay revealed that circGOLPH3 with wild-type miR-1299-binding site directly interacted with miR-1299 (Figure 3(j,k)). Next, the expression levels of miR-1299 in OSCC cells were examined. Compared with those in normal oral cells, the miR-1299 levels were downregulated in OSCC cells (Figure 3(l)). These results indicate that circGOLPH3 sponges miR-1299 and regulates its expression.

### CircGOLPH3 regulates the expression of LIF via miR-1299

**Figure 4.**
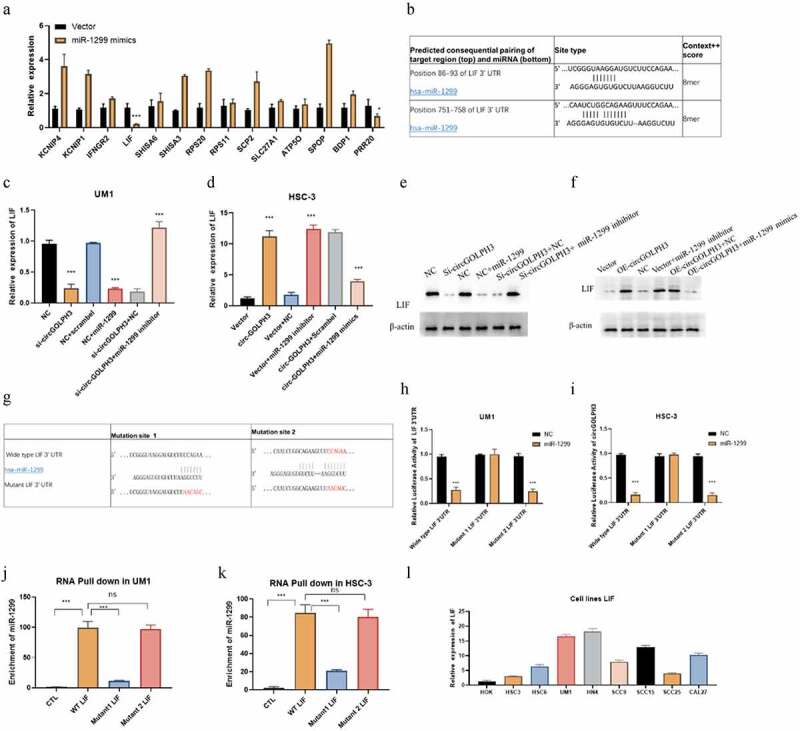
MicroRNA-1299 (miR-1299) regulates the expression of LIF. (a) UM1 cells were subjected to quantitative real-time polymerase chain reaction (qRT-PCR). (b) The predicted binding sites of miR-1299 in *LIF* 3’-untranslated region (UTR). (c) miR-1299 mimics were transfected into negative control (NC)-transfected UM1 cells, while miR-1299 inhibitor was transfected into si-circGOLPH3 UM1 cells. *LIF* mRNA levels in the transfected cells were examined using qRT-PCR. (d) miR-1299 inhibitor was transfected into empty vector-transfected HSC-3 cells, while miR-1299 mimics were transfected into circGOLPH3 overexpression plasmid-transfected HSC-3 cells. *LIF* mRNA levels in the transfected cells were determined using qRT-PCR. (e–f) LIF protein levels in UM1 (e) and HSC-3 (f) cells were examined using western blotting. (g) Mutants of two predicted miR-1299-binding sites in *LIF* 3’-UTR. (h–i) Luciferase activities of wild-type *LIF* 3’-UTR, *LIF* 3’-UTR with mutant binding site 1 and *LIF* 3’-UTR with mutant binding site 2. (j–k) UM1 and HSC-3 cells were subjected to RNA pull-down assay to determine the interaction of miR-1299 with wild-type *LIF* 3’-UTR, *LIF* 3’-UTR with mutant binding site 1, and *LIF* 3’-UTR with mutant binding site 2. (l) *LIF* mRNA levels in oral squamous cell carcinoma cell lines were examined using qRT-PCR.

MicroRNA is reported to regulate the expression of its target genes [33]. Therefore, the downstream targets of miR-1299 were examined. The target genes of miR-1299 were predicted using the bioinformatic tool Targetscan. The predicted results are shown in Supplementary Table 1. Most probable target genes were selected based on the cumulative weighted context++ score. mRNA levels of these target genes in miR-1299 mimic-transfected cells were determined using qRT-PCR (Figure 4(a)). *LIF* was determined to be a potential target gene of miR-1299 (Figure 4(a,b)). To confirm that *LIF* is a target of miR-1299, the mRNA and protein levels of LIF were determined using qRT-PCR and western blotting, respectively. circGOLPH3 knockdown downregulated the mRNA and protein levels of LIF. Additionally, transfection with miR-1299 mimics downregulated the expression of LIF (Figure 4(c,e)). Moreover, transfection with miR-1299 inhibitor mitigated the circGOLPH3 knockdown-mediated downregulation of LIF in UM1 cells (Figure 4(c,e)). circGOLPH3 overexpression or transfection with miR-1299 upregulated LIF levels in HSC-3 cells (Figure 4(d,f)). Transfection with miR-1299 mimics mitigated circGOLPH3 overexpression-induced upregulation of LIF (Figure 4(d,f)).

As Targetscan predicted two binding sites of miR-1299 in *LIF*, the exact binding site was determined by mutating the two predicted binding sites (Figure 4(g)). miR-1299 decreased the luciferase activities of wild-type *LIF* 3’-UTR and *LIF* 3’-UTR with mutant binding site 2 but not those of *LIF* 3’-UTR with mutant binding site 1 (Figure 4(h,i)). Next, RNA pull-down assay was performed to examine the interaction between miR-1299 and *LIF* 3’-UTR. Binding site 1 mutation effectively inhibited the binding between miR-1299 and *LIF* 3’-UTR, whereas binding site 2 mutation did not affect the binding (Figure 4(j,k)). qRT-PCR analysis revealed that the mRNA expression levels of *LIF* were upregulated in OSCC cells (Figure 4(l)). These findings indicated that circGOLPH3 regulates LIF expression through miR-1299 in OSCC.

### CircGOLPH3\miR-1299\LIF axis regulates OSCC progression

**Figure 5.**
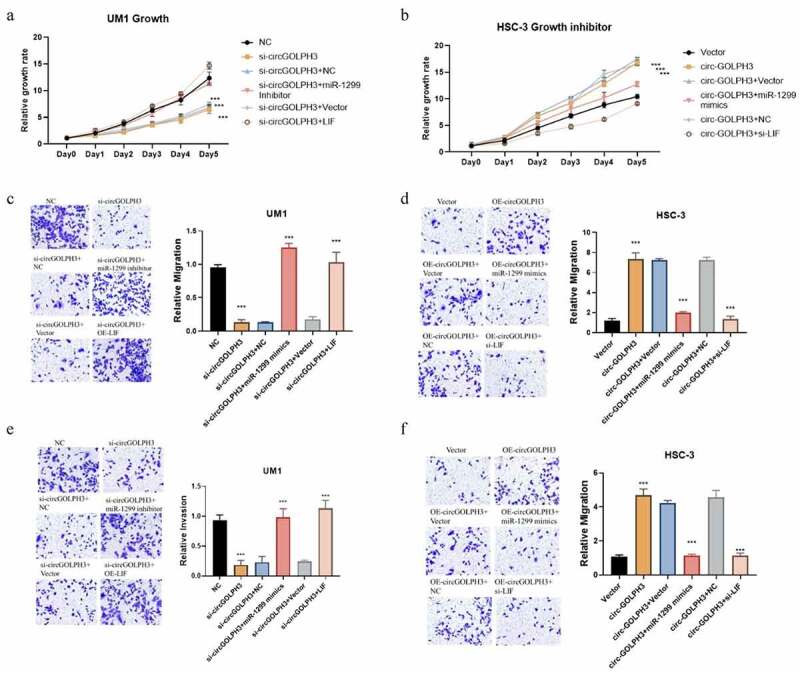
Circular *GOLPH3* RNA (circGOLPH3)\miR-1299\LIF axis promotes the proliferation, migration, and invasion of oral squamous cell carcinoma cells. (a) si-circGOLPH3-transfected UM1 cells were transfected with miR-1299 inhibitor or LIF overexpression plasmids. The proliferation rates of transfected cells were examined using the cell counting kit 8 (CCK8) assay. (b) miR-1299 mimics or short-interfering RNAs (siRNAs) targeting *LIF* were transfected into circGOLPH3-overexpressing HSC-3 cells. The proliferation rates of transfected cells were determined using the CCK8 assay. (c–d) Migration of UM1 (c) and HSC-3 cells (d) was examined using the migration assays. (e–f) Invasion of UM1 (e) and HSC-3 cells (f) was examined using the invasion assays.

The role of the miR-1299\LIF pathway in circGOLPH3-mediated OSCC progression was examined. circGOLPH3 knockdown UM1 cells were transfected with miR-1299 inhibitor and LIF expression plasmids. Meanwhile, circGOLPH3-overexpressing HSC-3 cells were transfected with miR-1299 mimics and si-LIF. Transfection with miR-1299 inhibitor and LIF expression plasmids mitigated the circGOLPH3 knockdown-induced downregulation of UM1 cell proliferation, migration, and invasion (Figure 5(a,c,e)). Meanwhile, transfection with miR-1299 mimics and si-LIF mitigated the circGOLPH3 overexpression-induced upregulation of HSC-3 cell proliferation, migration, and invasion (Figure 5(b,d,f)). These results indicate that circGOLPH3 regulates OSCC progression through the miR-1299\LIF pathway.

### *Oncogenic activity of circGOLPH3* in vivo

**Figure 6.**
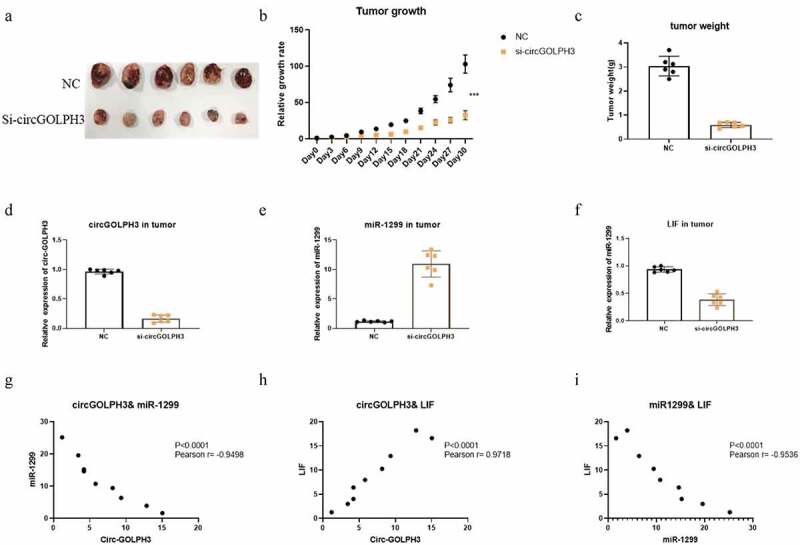
circGOLPH3\miR-1299\LIF axis in vivo. a. Nude mice were subcutaneously injected with circGOLPH3 stably silenced UM1 cells and UM1 NC cells. 30 days later, the tumors were collected and photographed. b. The relative growth rates of tumors in nude mice. c. Relative weights of tumors in nude mice. d-f. The expressions of circGOLPH3 (d), miR-1299(e), and LIF (f) were detected by qRT-PCR. g. The correlation between circGOLPH3 and miR-1299 in OSCC cell lines. h. The correlation between circGOLPH3 and LIF in OSCC cell lines. i.The correlation between LIF and miR-1299 in OSCC cell lines.

Next, the role of circGOLPH3 *in vivo* was examined. Nude mice were subcutaneously implanted with negative control (NC)-transfected or si-circGOLPH3-transfected UM1 cells. The growth of the tumor derived from si-circGOLPH3-transfected cells was suppressed in nude mice (Figure 6(a,b)). The weight of the tumor derived from si-circGOLPH3-transfected UM1 cells was lower than that of the tumor derived from NC-transfected cells (Figure 6(c)). This indicates that circGOLPH3 promotes OSCC cell proliferation *in vivo*. qRT-PCR analysis revealed that the tumor derived from si-circGOLPH3-transfected UM1 cells (Figure 6d) exhibited upregulated miR-1299 expression (Figure 6e) and downregulated LIF expression (Figure 6(f)), which was consistent with the results of *in vitro* experiments.

Further, the correlation between circGOLPH3, miR-1299, and LIF was examined. The expression of circGOLPH3 was negatively correlated with that of miR-1299 (Figure 6(g)) but positively correlated with that of LIF (Figure 6(h)) in OSCC cell lines. Additionally, the expression of miR-1299 was negatively correlated with that of LIF in OSCC cell lines (Figure 6(i)). These results suggest the important role of the circGOLPH3\miR-1299 \LIF axis in OSCC progression.

## Discussion

circRNA, a type of noncoding RNA exhibiting loop structure and enhanced stability, is widely expressed in various types of tissues. Previous studies have reported that circRNAs are involved in the pathogenesis of cancer. circRNAs exert their effects through various mechanisms, including microRNA sponging mechanisms. The sponging of microRNAs by circRNA inhibits the regulatory effects of microRNAs on their target genes [34]. The elucidation of the mechanism underlying cancer progression will provide valuable information for the development of cancer therapy.

The expression and function of circGOLPH3 in OSCC have not been previously examined. In this study, we determined the expression levels of circGOLPH3 in OSCC cells and reported that circGOLPH3 is mainly localized to the cytoplasm (Figure 1(a-d)). The characteristics of circGOLPH3 were determined using the RNase R and RNA half-time assays (Figure 1(g,f)). circGOLPH3 was stable and resistant to RNase R-mediated digestion. qRT-PCR analysis revealed that the expression levels of circGOLPH3 in OSCC cells were higher than those in non-cancerous cells (Figure 1(i)). Functional experiments revealed that circGOLPH3 promotes the proliferation, migration, and invasion of OSCC cells (Figure 2).

MicroRNAs, which are a subset of small noncoding RNAs with a length of 19–24 nucleotides [35], regulate gene expression at the post-transcriptional level by targeting the 3’-UTR of mRNA, which leads to the degradation of mRNA. miR-1299, which is reported to function as a tumor suppressor in various cancers [35–37], is downregulated in several cancers [38–41]. However, the function of miR-1299 in OSCC has not been previously investigated. This study demonstrated that circGOLPH3 is a novel regulator of miR-1299, which provided useful insights into OSCC progression. Future studies must investigate circGOLPH3-mediated regulation of miR-1299 in other cancers.

This study also identified the downstream target of miR-1299 (Figure 3). The results of the qRT-PCR, RNA pull-down, and luciferase assays revealed that miR-1299 sponges *LIF* mRNA by directly interacting with binding site 1 of *LIF* 3’-UTR (Figure 4). LIF, a member of the IL6 family [42], is involved in several pathological and physiological processes, including proliferation, regeneration, infection, inflammation, and immune response. Previous studies have reported that LIF exerts oncogenic effects in several solid cancers [43]. Thus, LIF is a novel molecular target for various cancers. In patients with OSCC, the upregulated expression of LIF is correlated with poor survival [44]. However, the mechanism underlying the regulation of LIF in cancer has not been elucidated. This study demonstrated that circGOLPH3 regulates LIF expression through miR-1299. The detailed elucidation of the mechanism underlying LIF regulation will aid in the development of targeted therapies for OSCC.

Based on these previous studies, a series of experiments was performed to investigate the role of circGOLPH3 in OSCC progression. circGOLPH3 exhibited oncogenic activities during OSCC progression *in vitro*. Mechanistically, circGOLPH3 sponges miR-1299 and consequently inhibits the regulatory effects of miR-1299 on its target gene *LIF*. The binding sites of miR-1299 in circGOLPH3 and *LIF* were identified in this study. Inhibition of the circGOLPH3\miR-1299\LIF axis suppressed the migration, invasion, and proliferation of OSCC cells (Figure 5). The results of *in vivo* experiments also demonstrated that circGOLPH3 promoted OSCC cell proliferation (Figure 6). This study is associated with some limitations. All experiments in this study were performed in OSCC cell lines and nude mice. Additionally, the upstream regulatory mechanism of circGOLPH3 and the specific mechanisms underlying the LIF-mediated tumorigenesis of OSCC were not elucidated. The function of circGOLPH3 in clinical samples will be examined in future studies.

## Conclusion

This study determined the important roles of circGOLPH3 in OSCC cell proliferation, migration, and invasion. Inhibition of the circGOLPH3\miR-1299\LIF axis effectively suppressed OSCC cell proliferation, migration, and invasion. Thus, circGOLPH3 is a potential novel diagnostic or therapeutic biomarker for OSCC.

## Supplementary Material

Supplemental Material

## Data Availability

The data of this study are available from the corresponding author upon reasonable request.
