# Supplementary material for: Circular GOLPH3 RNA exerts oncogenic effects in vitro by regulating the miRNA-1299/LIF axis in oral squamous cell carcinoma
Source: Bioengineered. 2022 Apr 28;13(4):11012–25. doi: 10.1080/21655979.2022.2067288 (PMC9208457; doi:10.1080/21655979.2022.2067288)
Supplement: Supplemental Material [file KBIE_A_2067288_SM7788.zip › supplementary/microscopy images including original scale.docx]

**Figure 2M**

UM1 NC migration


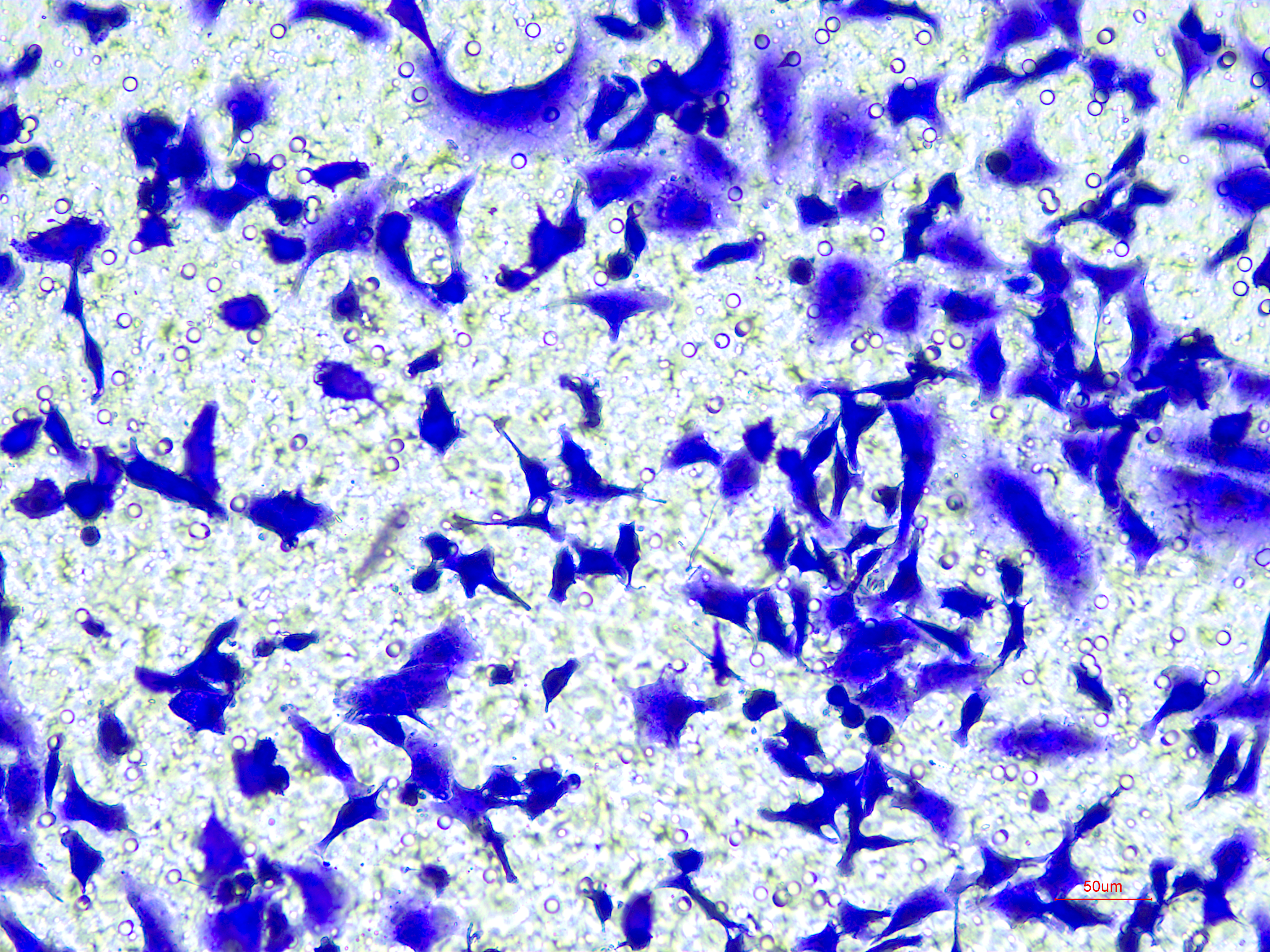


UM1 si-circGOLPH3 migration


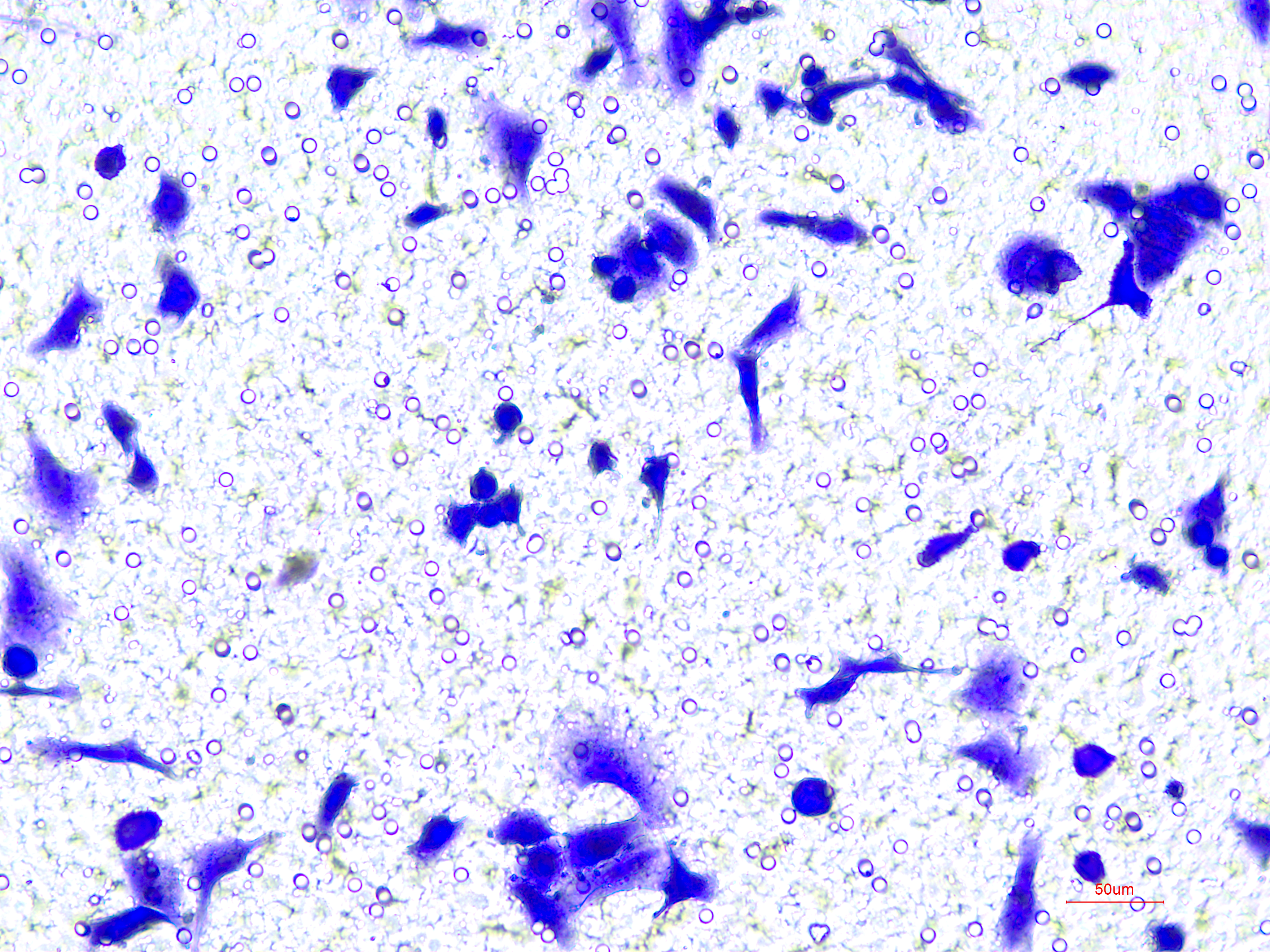


**Figure2O**

**HSC-3 Vector Migration**

**
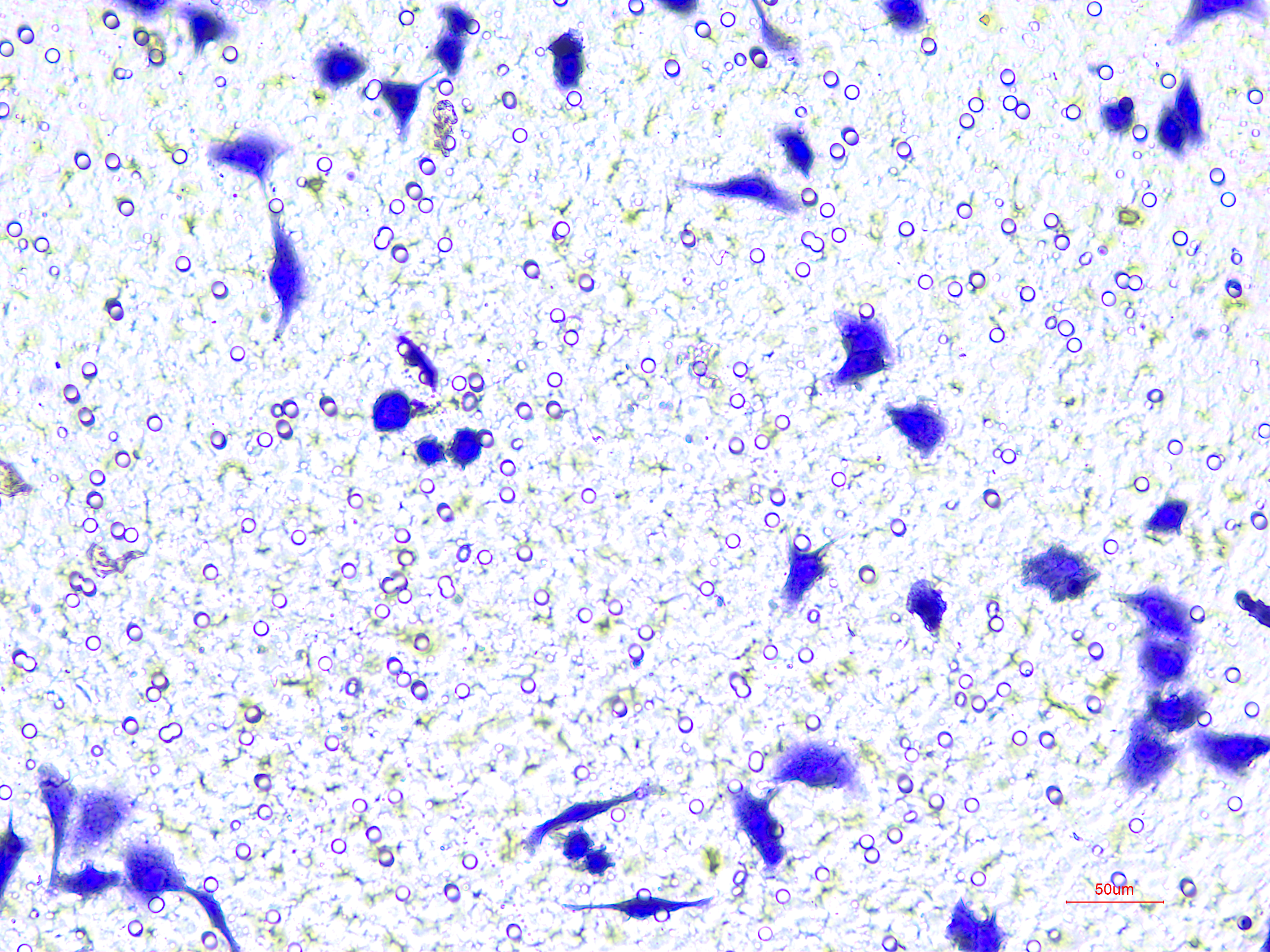
**

**HSC-3 OE-circGOLPH3 Migration**

**
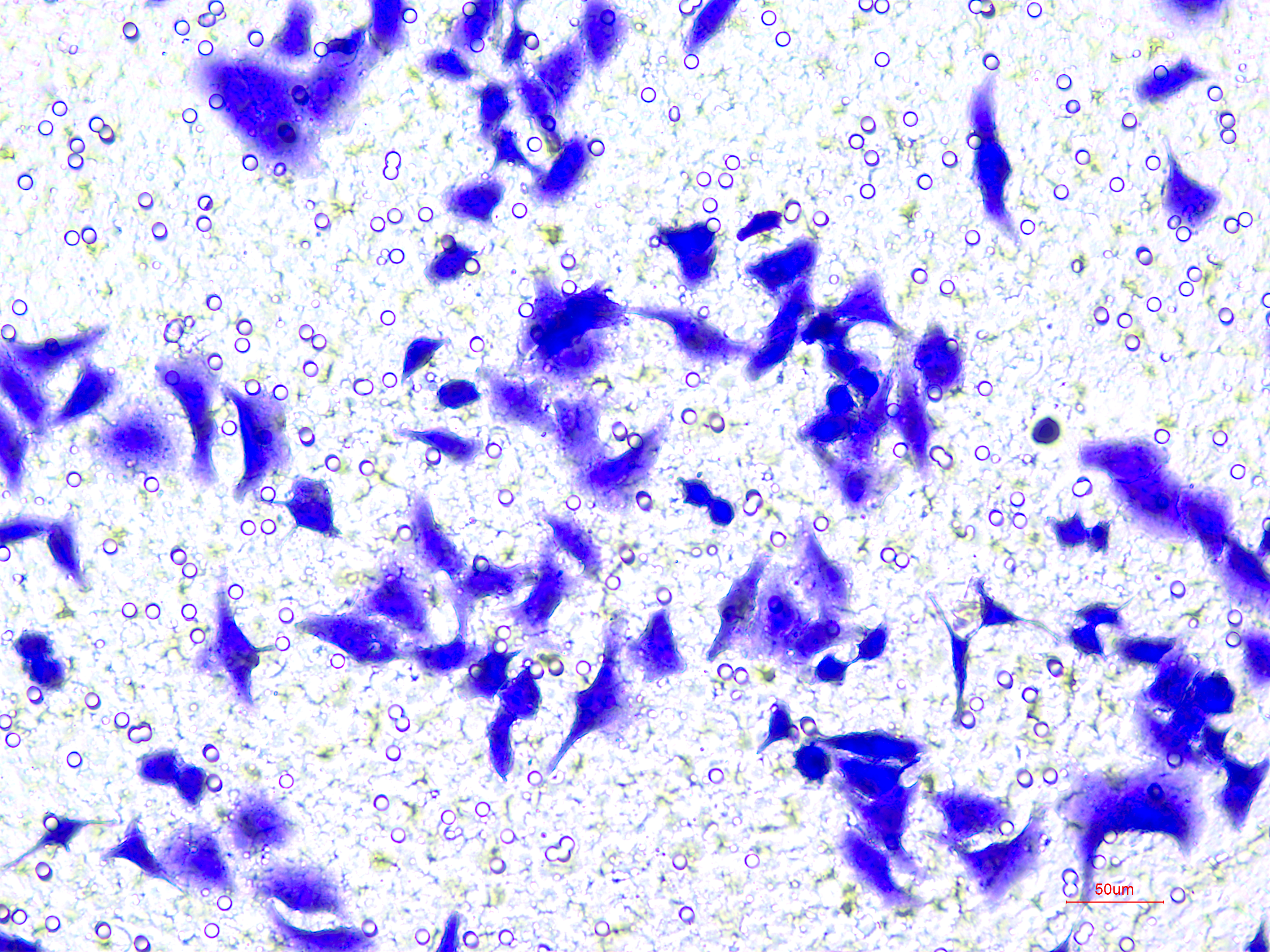
**

**Figur2Q**

**UM1 NC Invasion**

**
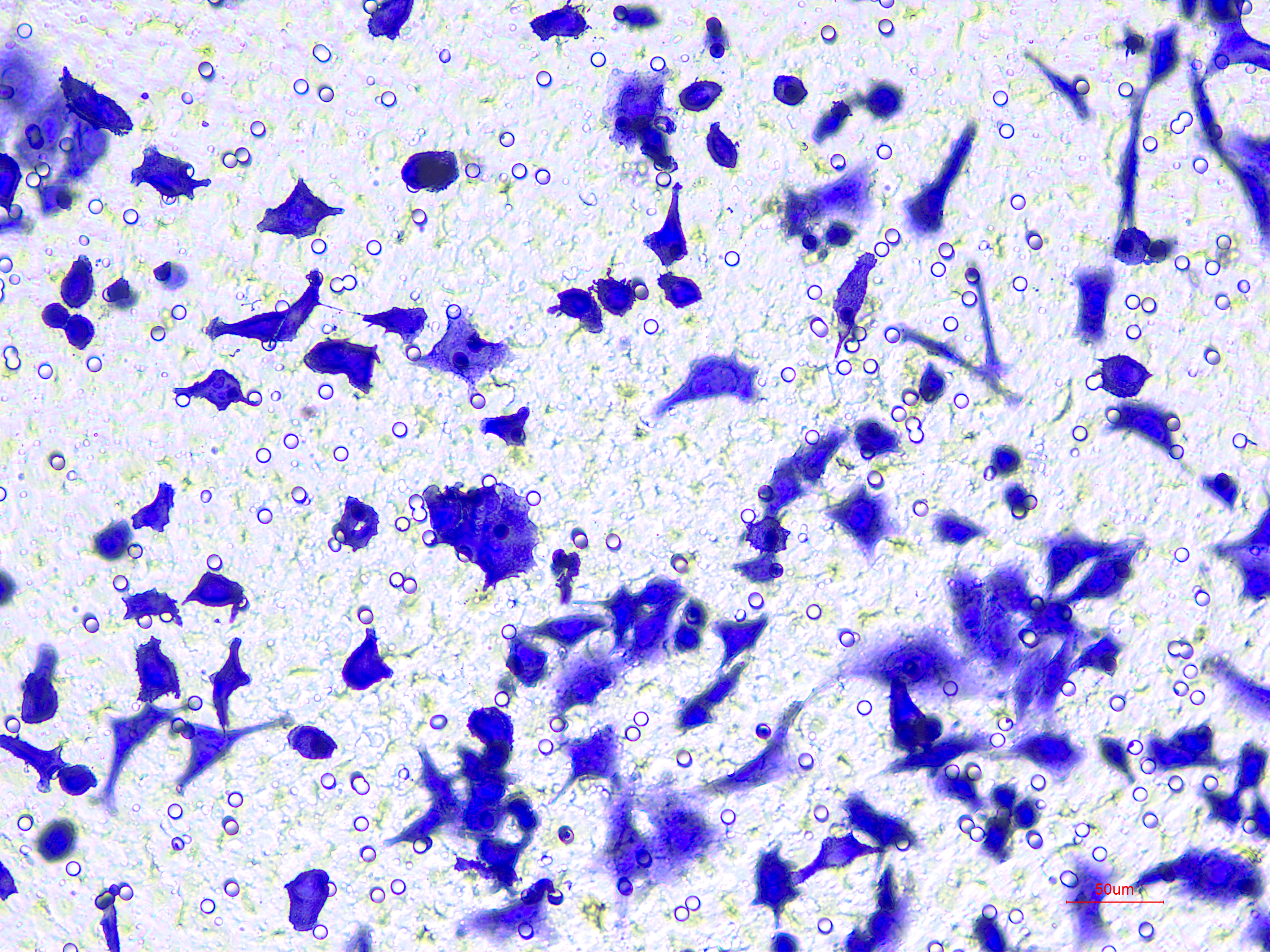
**

**UM1 si-circGOLPH3 Invasion**

**
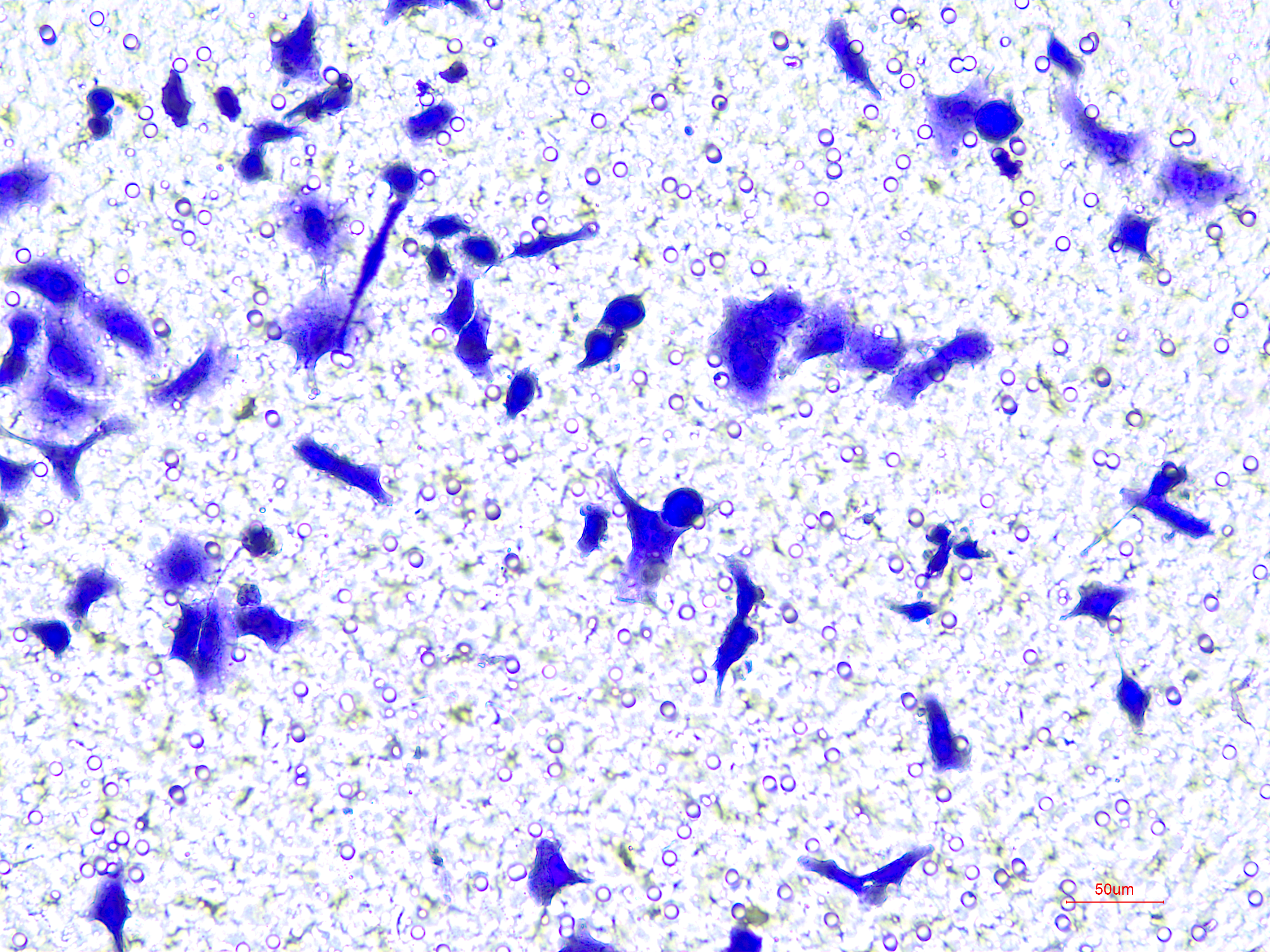
**

**Figure 2S**

**HSC3 Vector invasion**

**
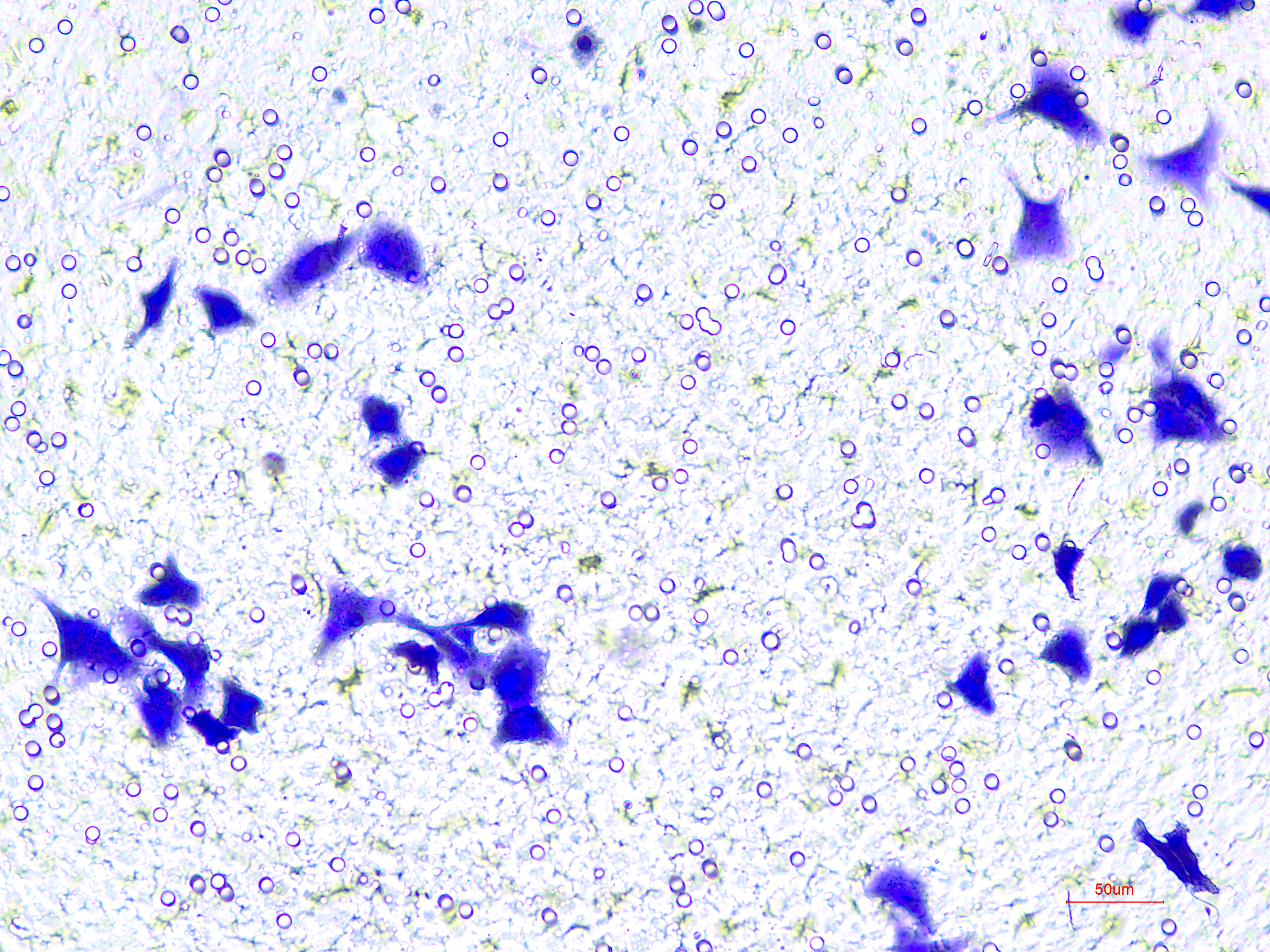
**

**HSC3 OE-circGOLPH3 Invasion**

**
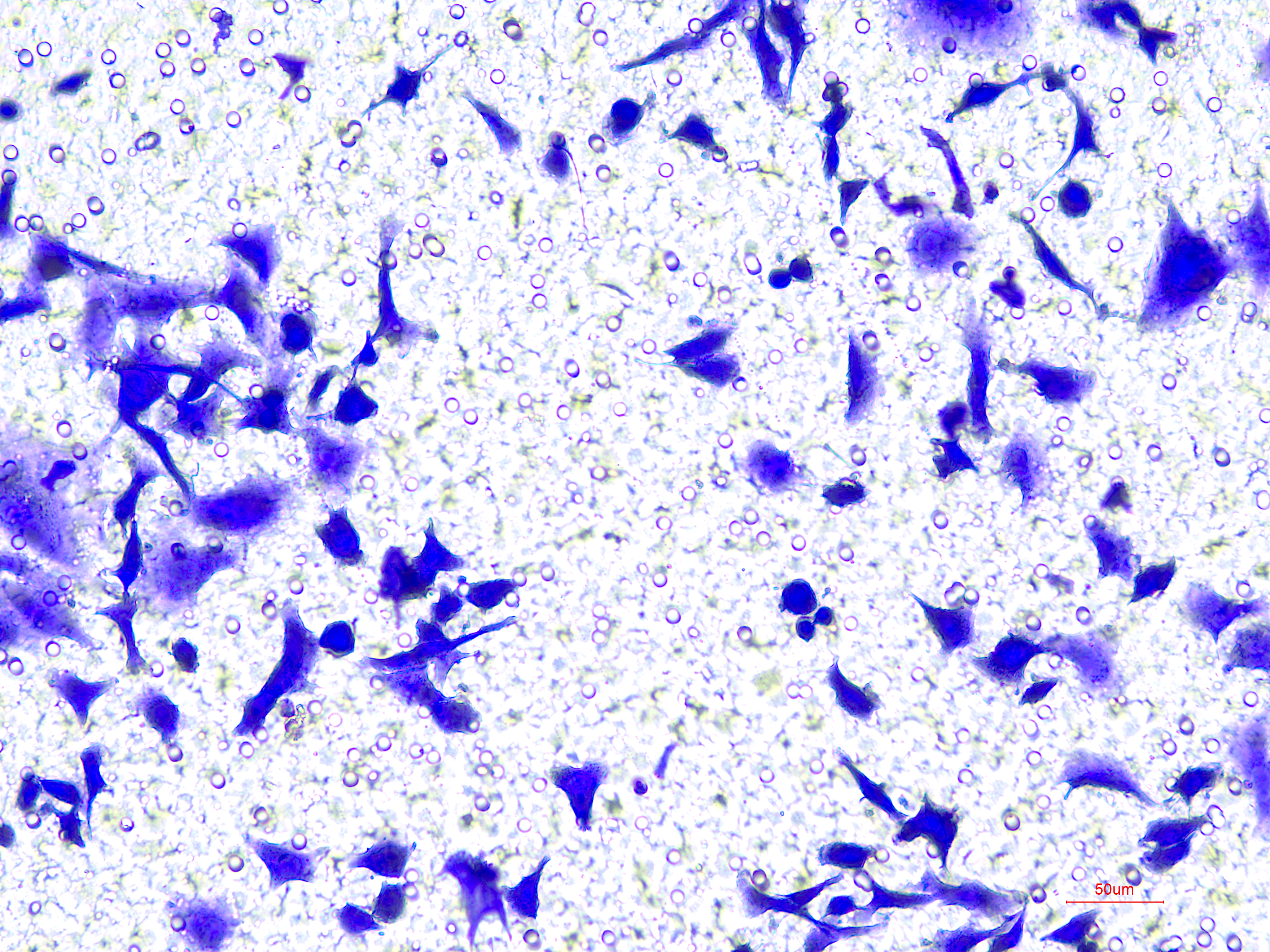
**

**Figure 5C**

**UM NC Migration**

**
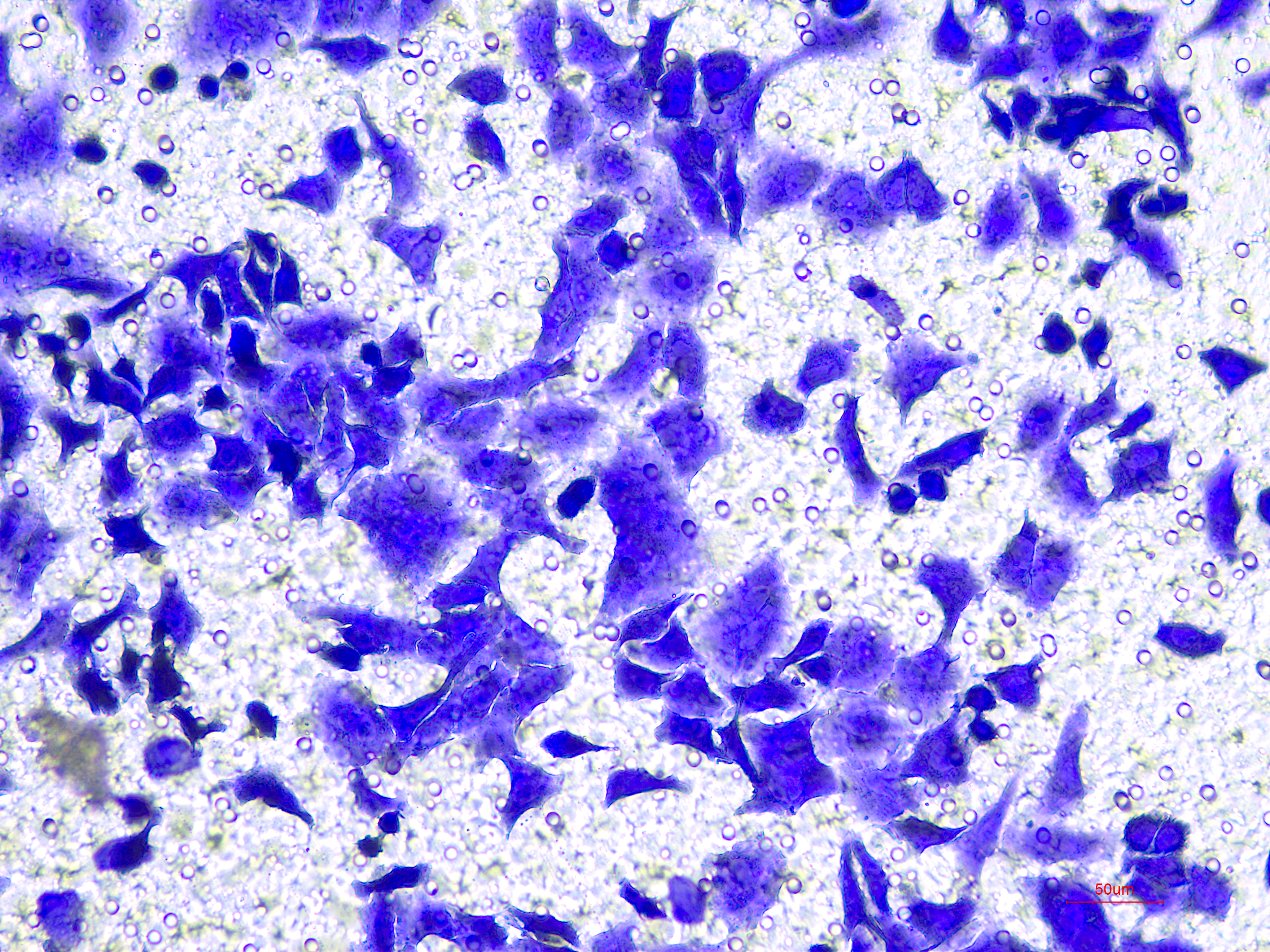
**

**UM1 si-circGOLPH3 Migration**

**
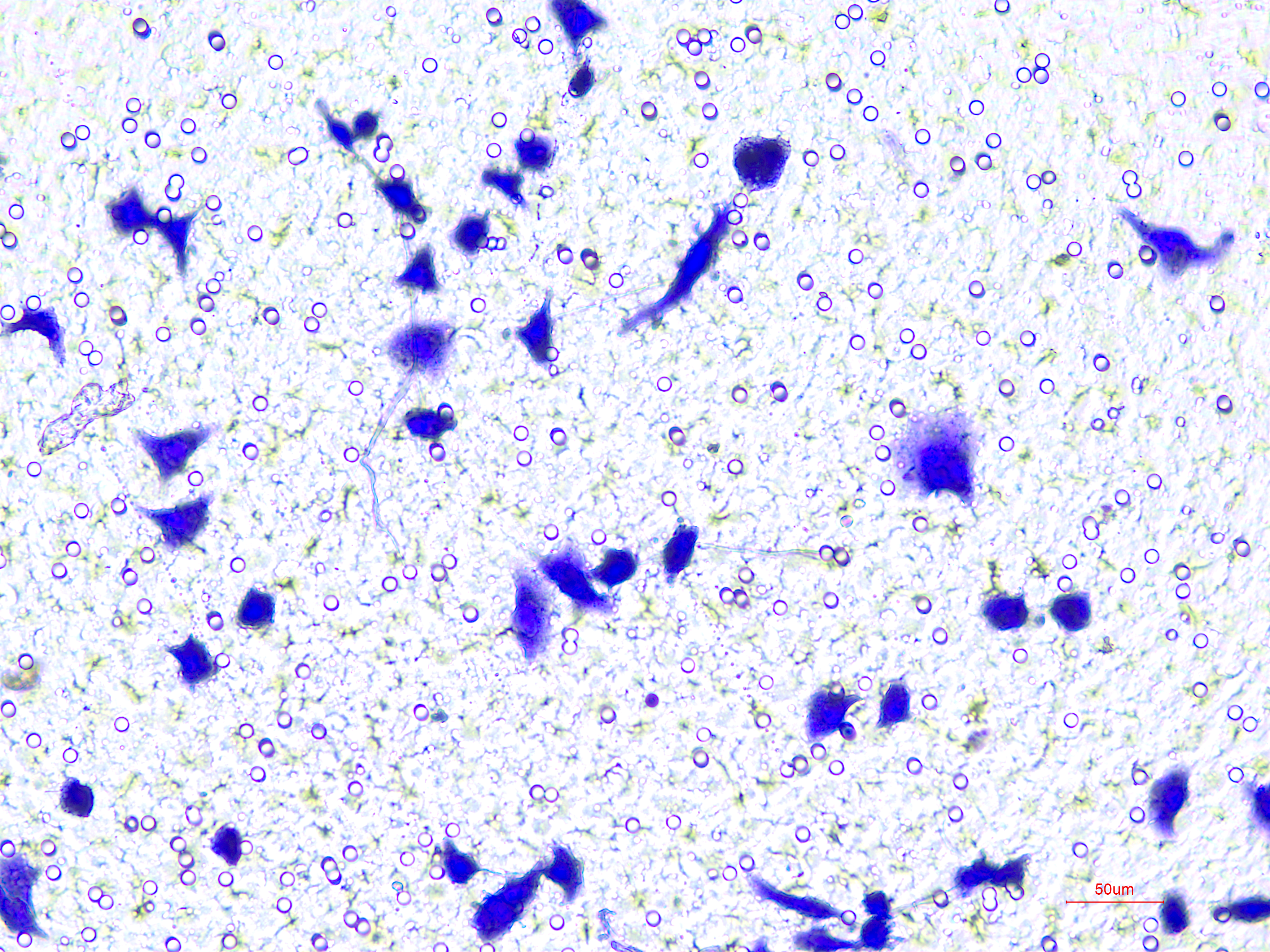
**

**UM1si-circGOLPH3+NC migration**

**
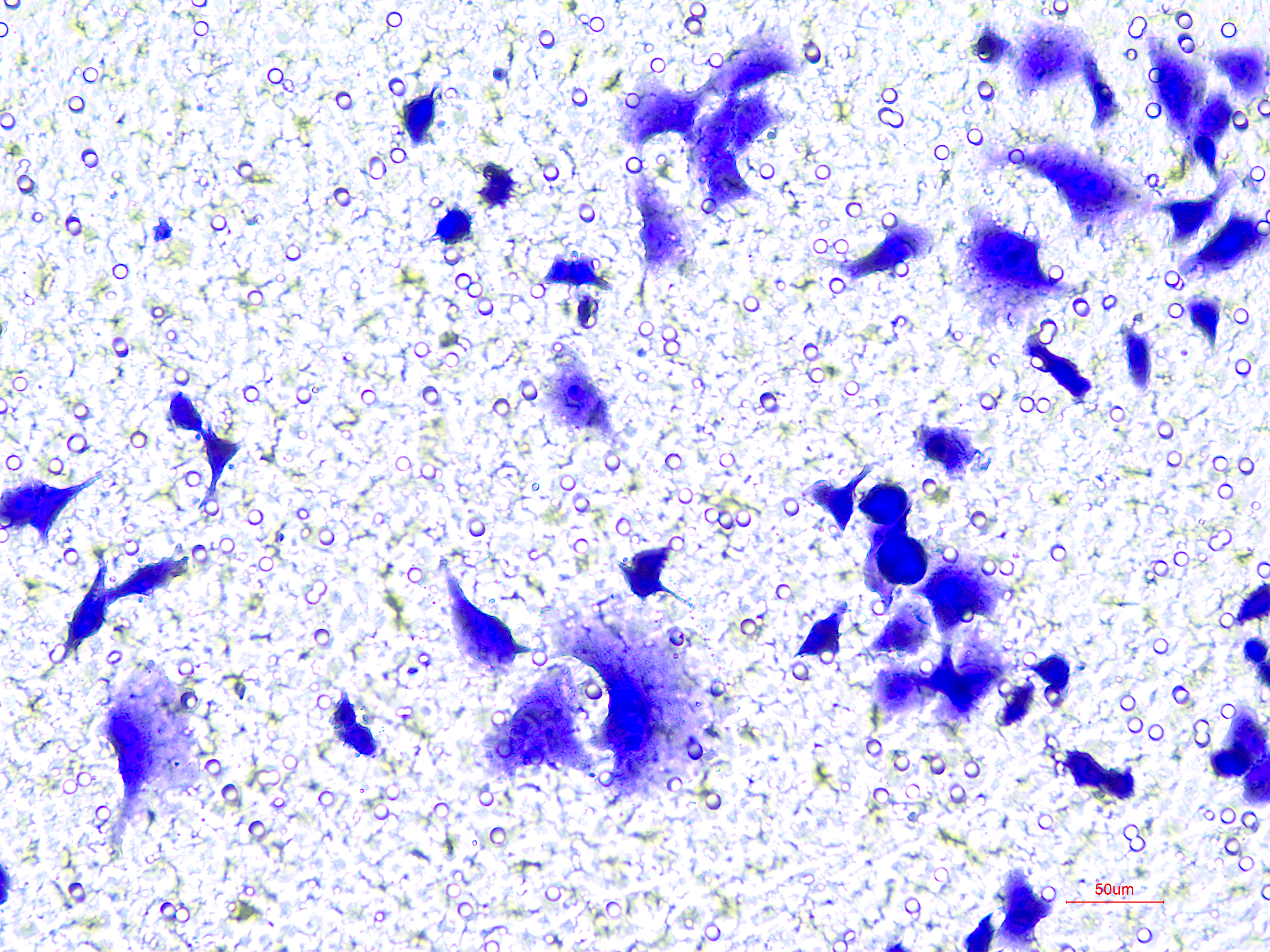
**

**UM1 si-circGOLPH3+miR1299 inhibitor migration**

**
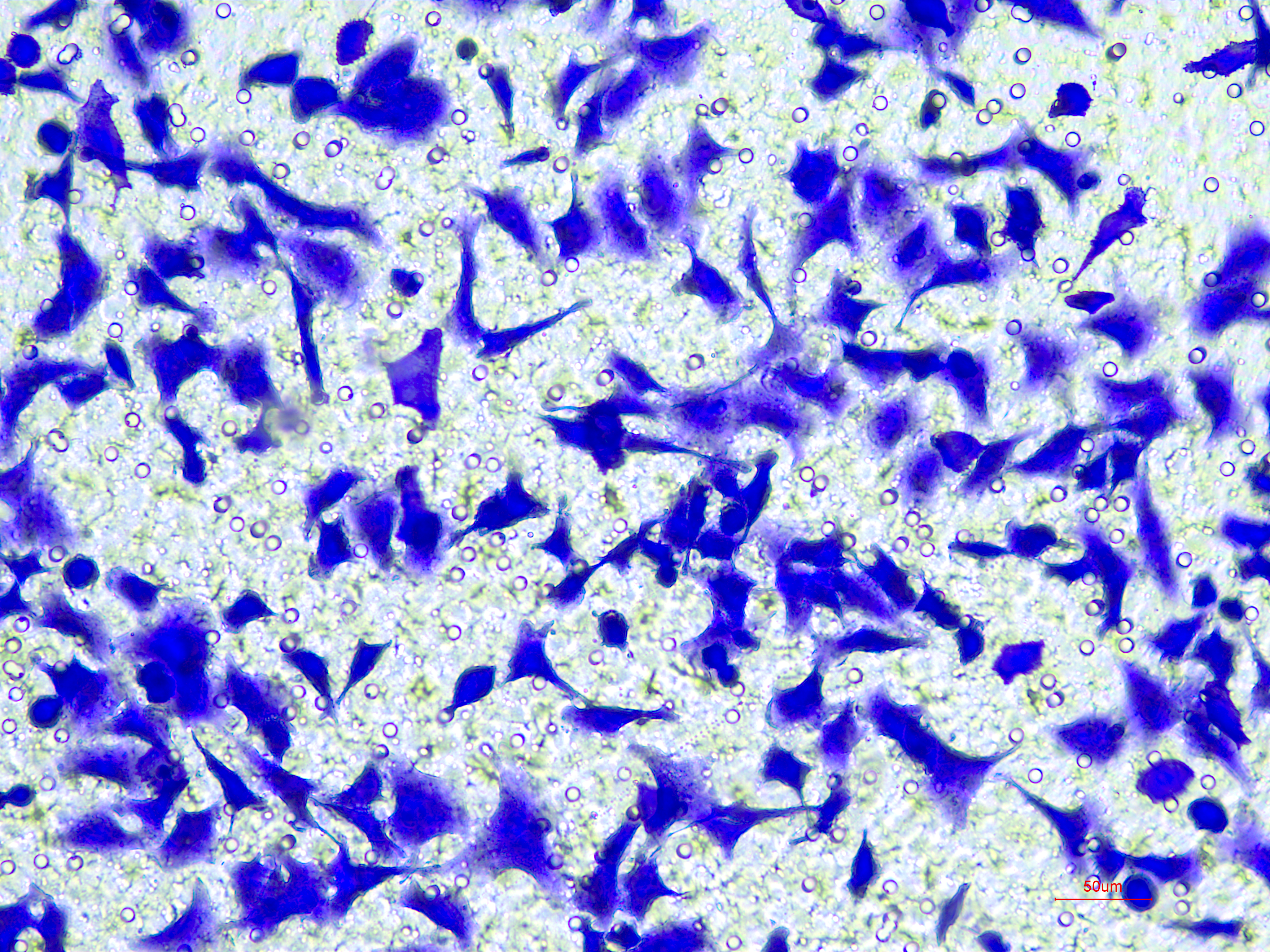
**

**UM1 si-circGOLPH3 +Vector migration**

**
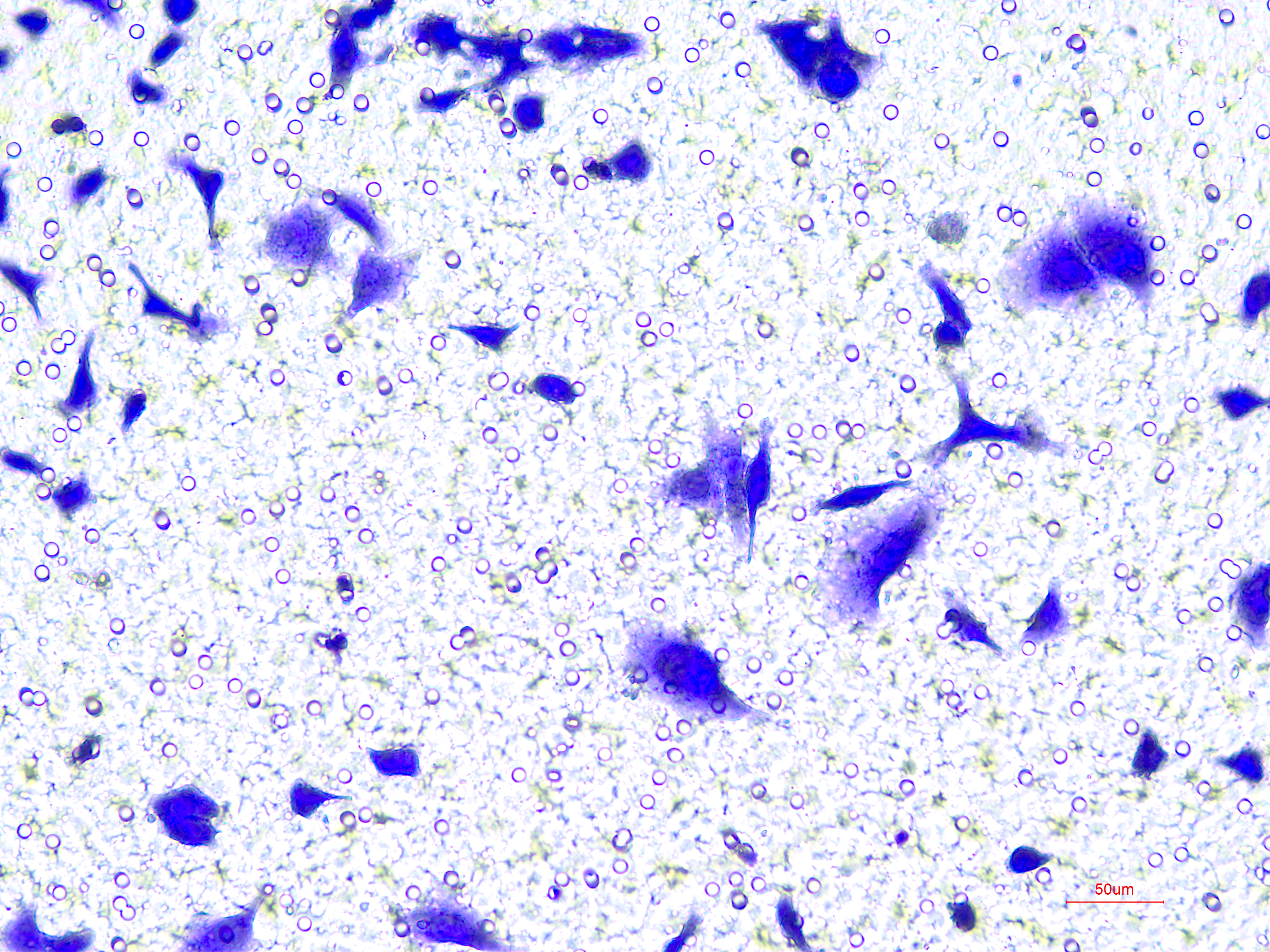
**

**UM1 si-circGOLPH3+OE-LIF migration**

**
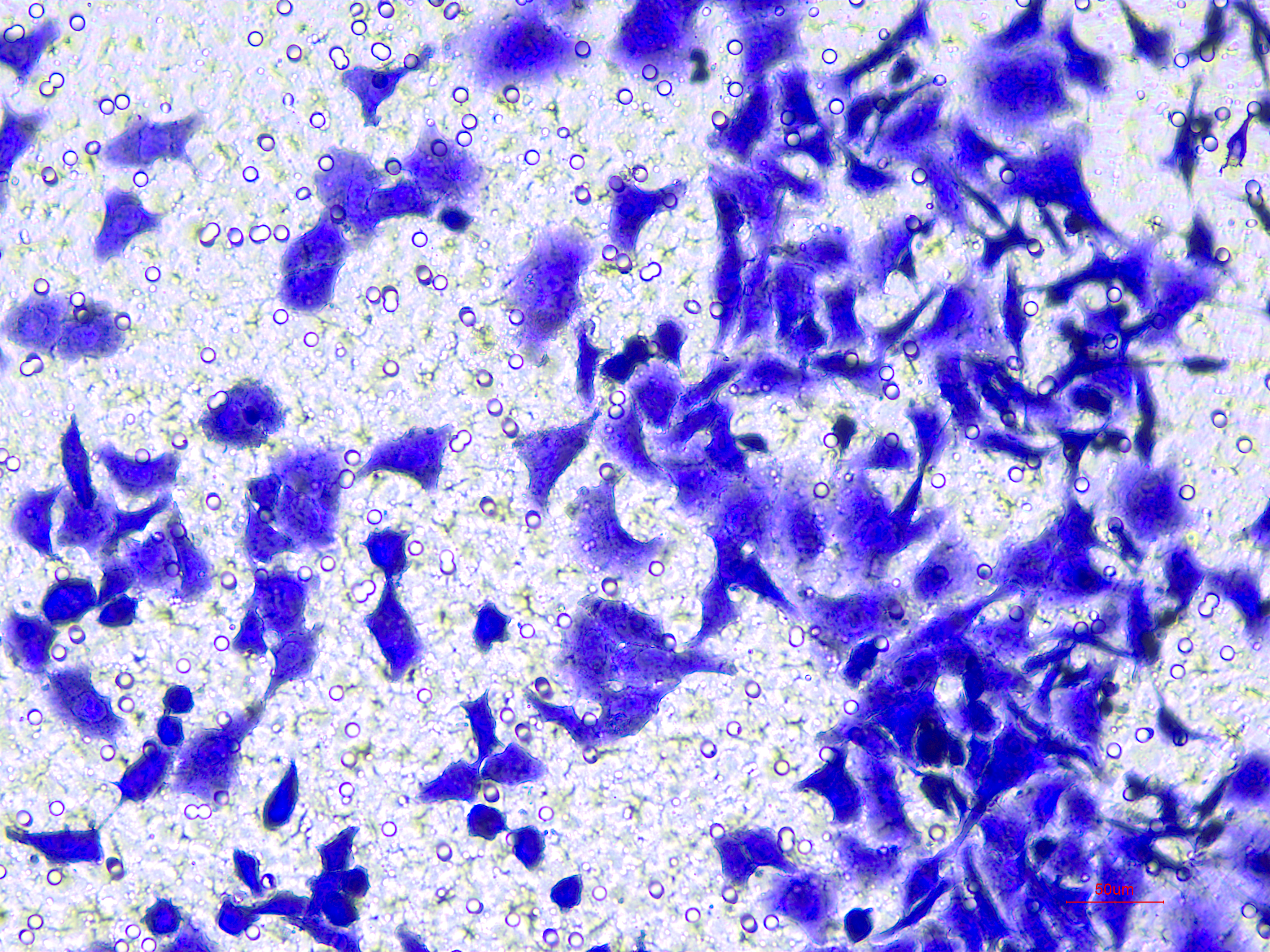
**

**Figure 5D**

**HSC Vector migration**

**
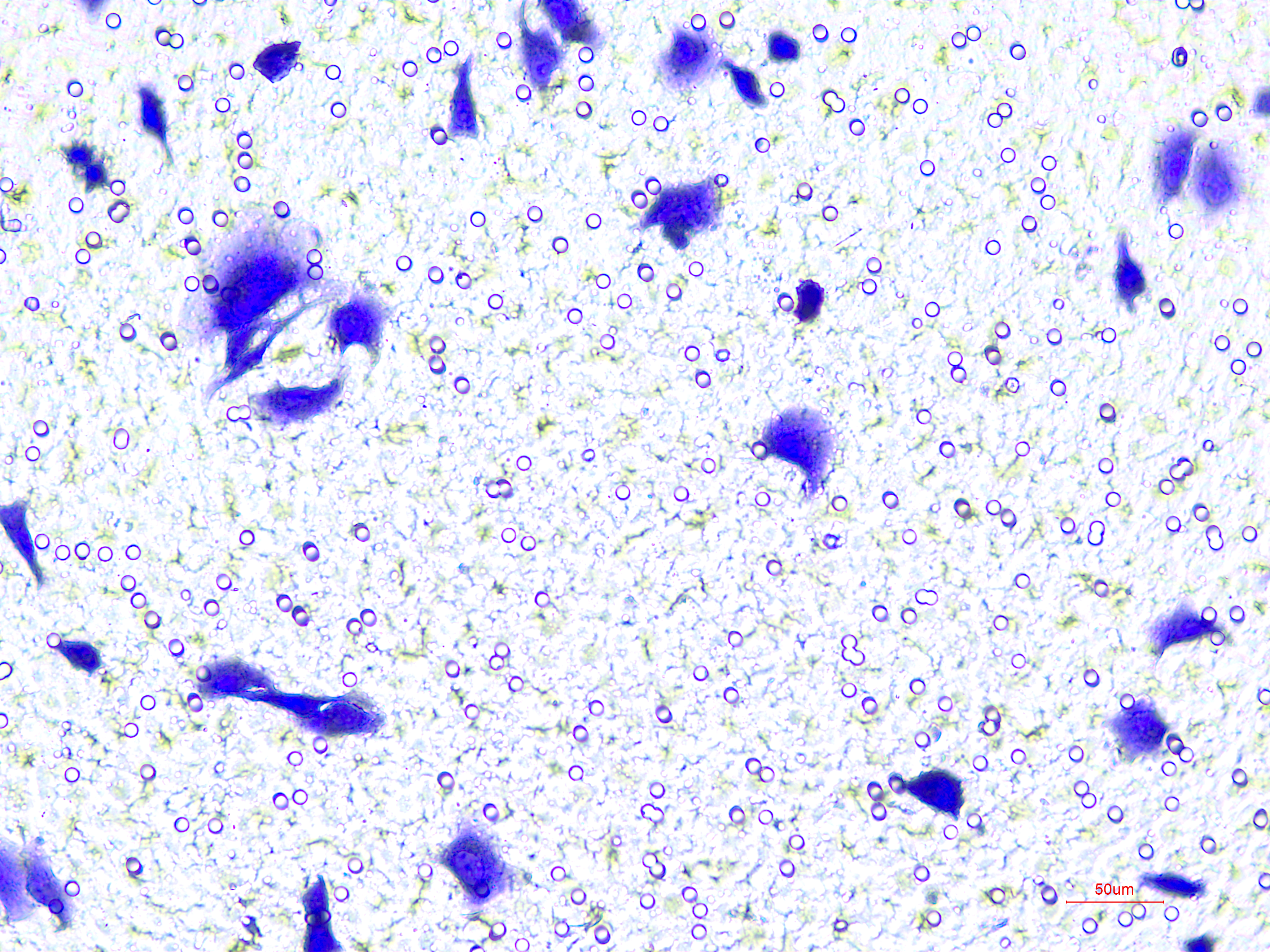
**

**HSC3 OE-circGOLPH3 migration**

**
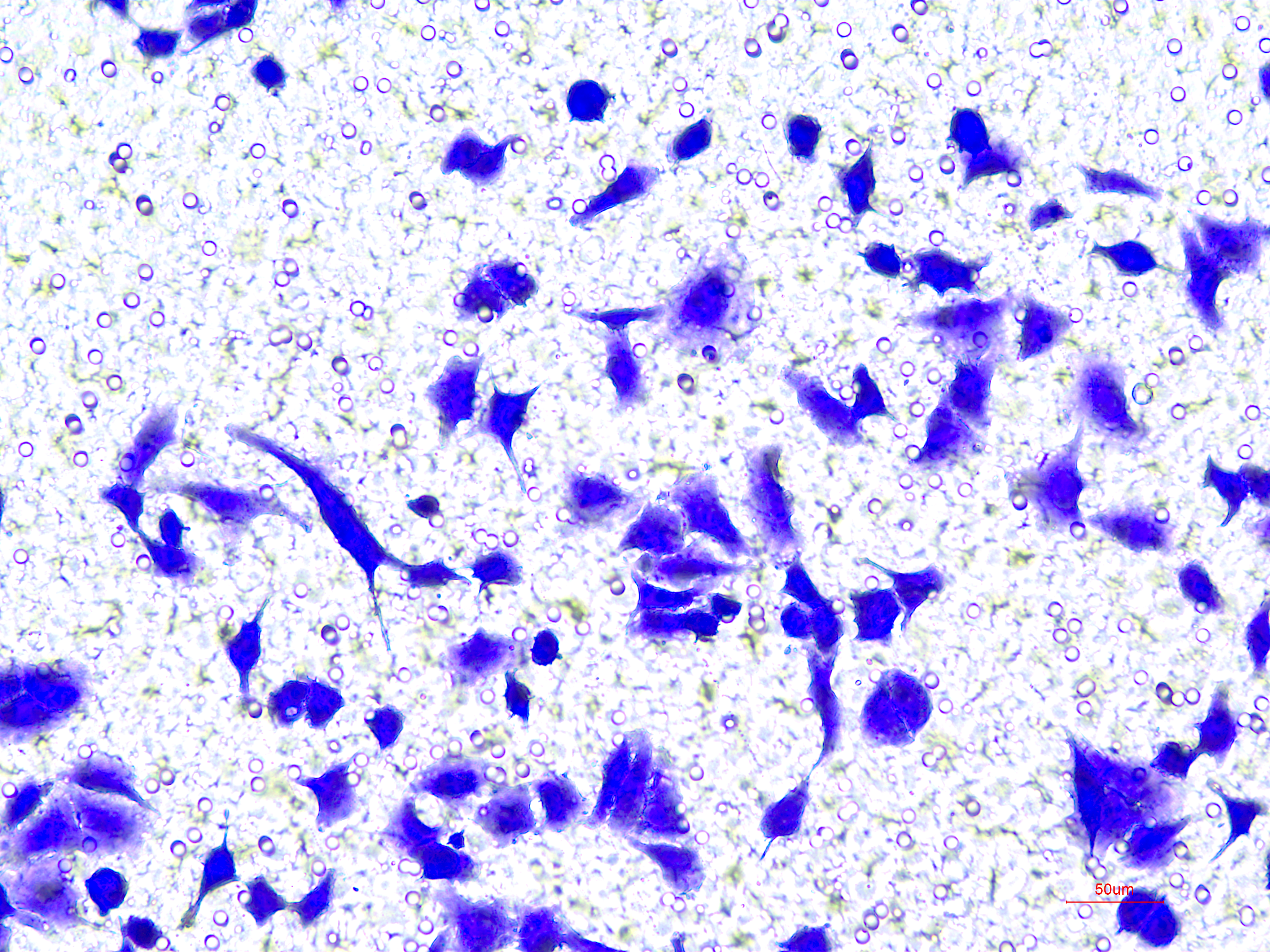
**

**HSC3 OE-circGOLPH2+Vector migration**

**
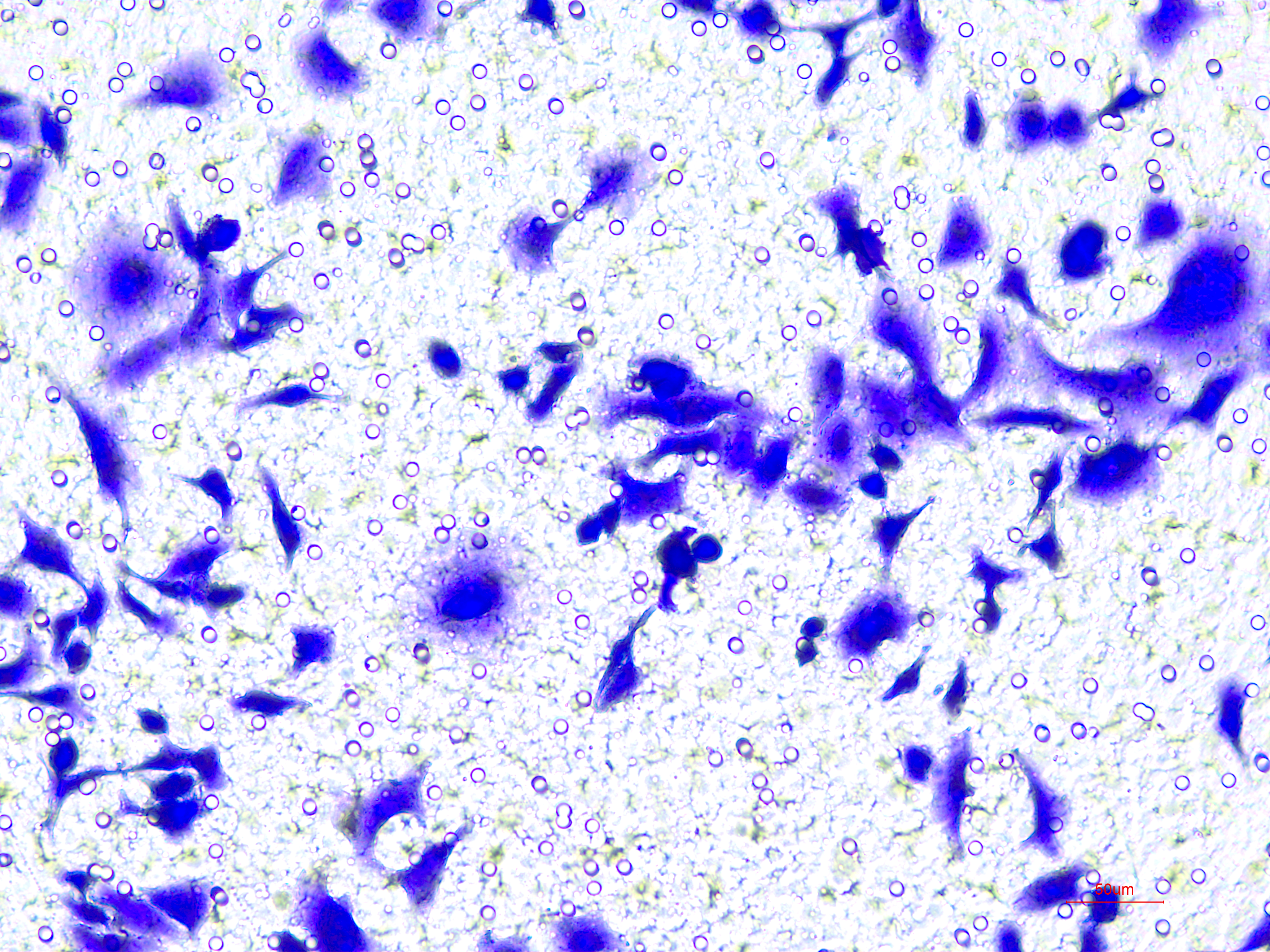
**

**HSC3 OE-circGOLPH3 +miR1299 mimics**

**
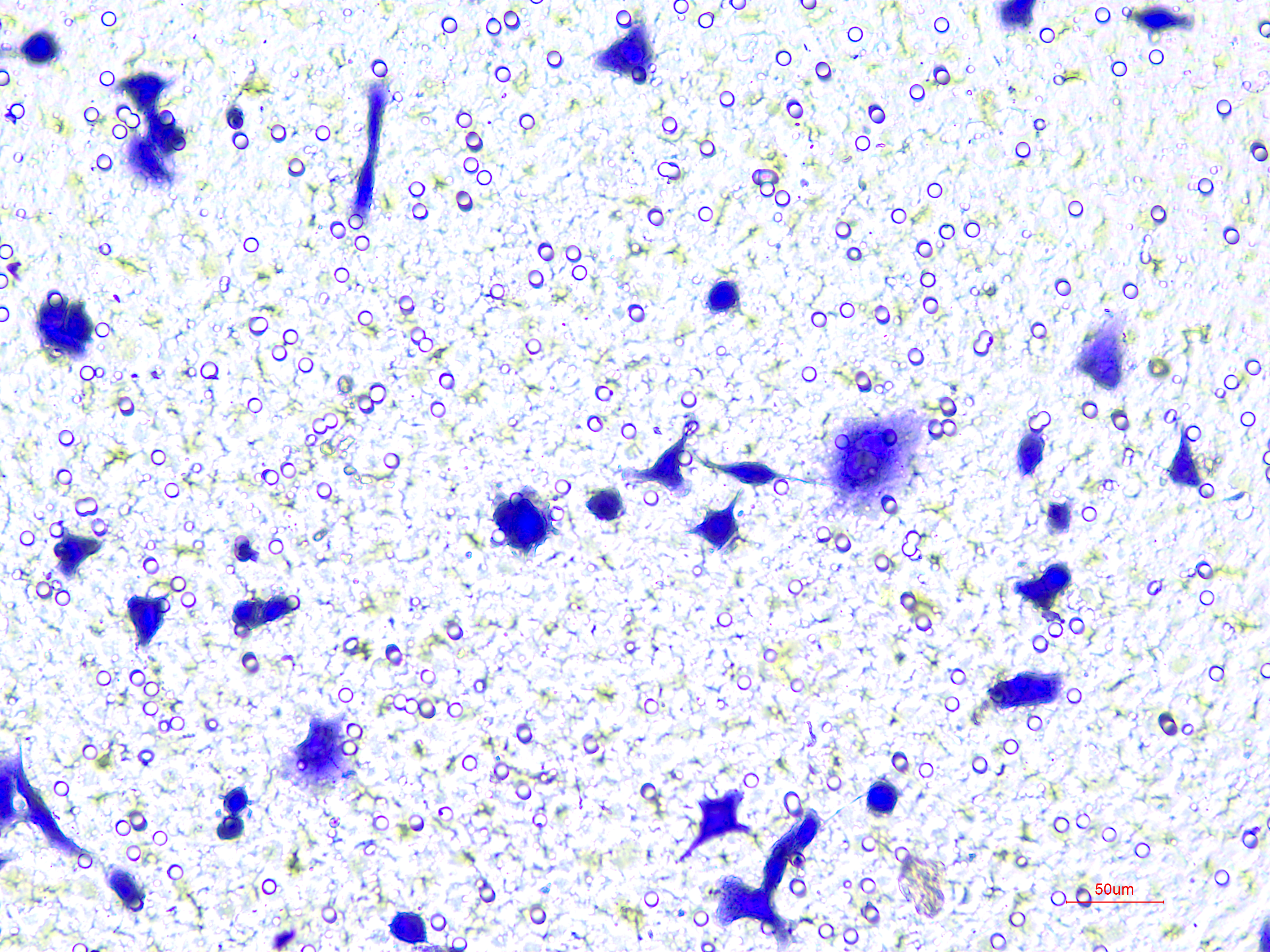
**

**HSC3 OE-circGOLPH3 +NC migration**

**
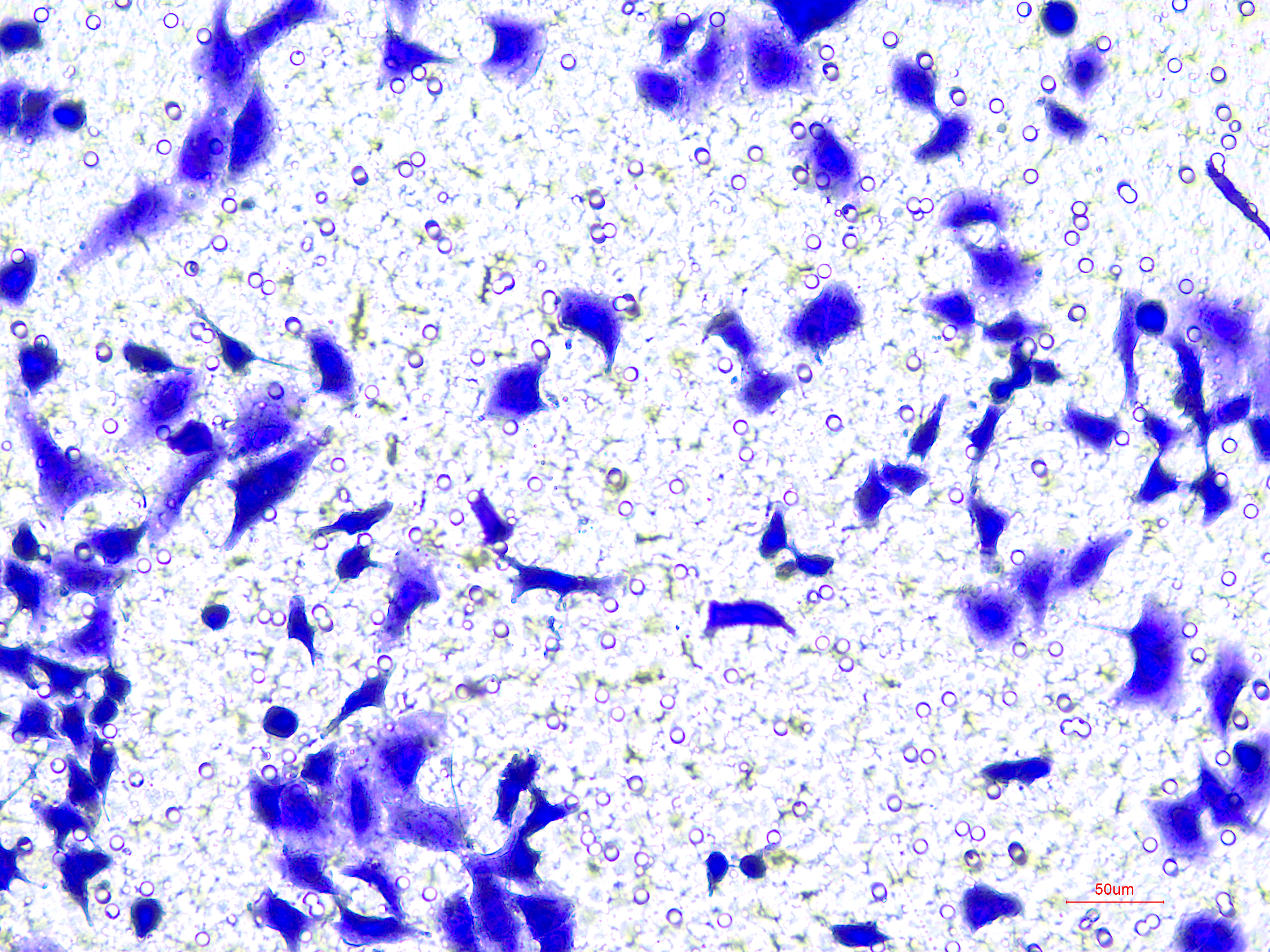
**

**HSC3 OE-circGOLPH3 +si-LIF migration**

**
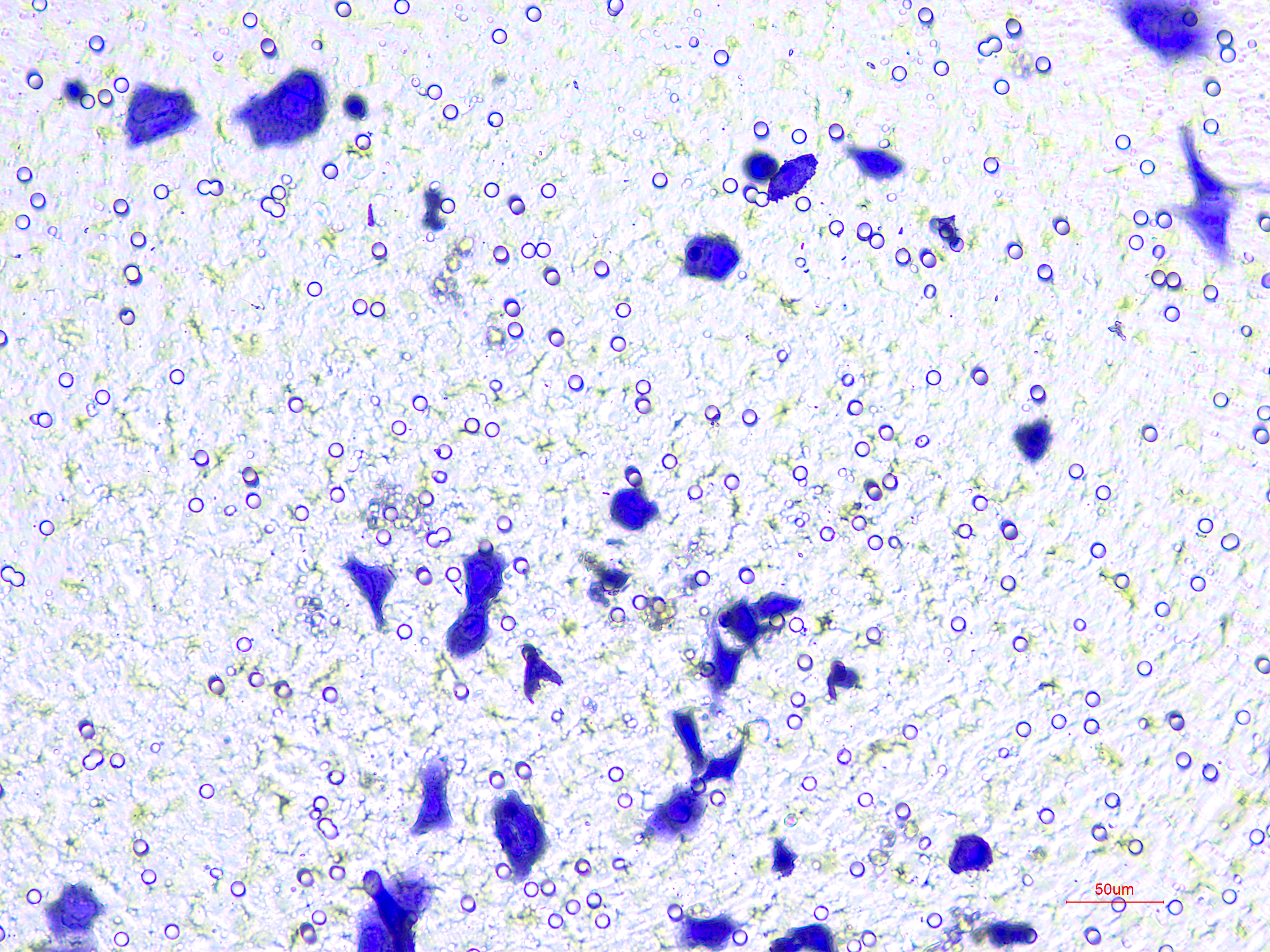
**

**Figure5E**

**UM1 NC Invasion**

**
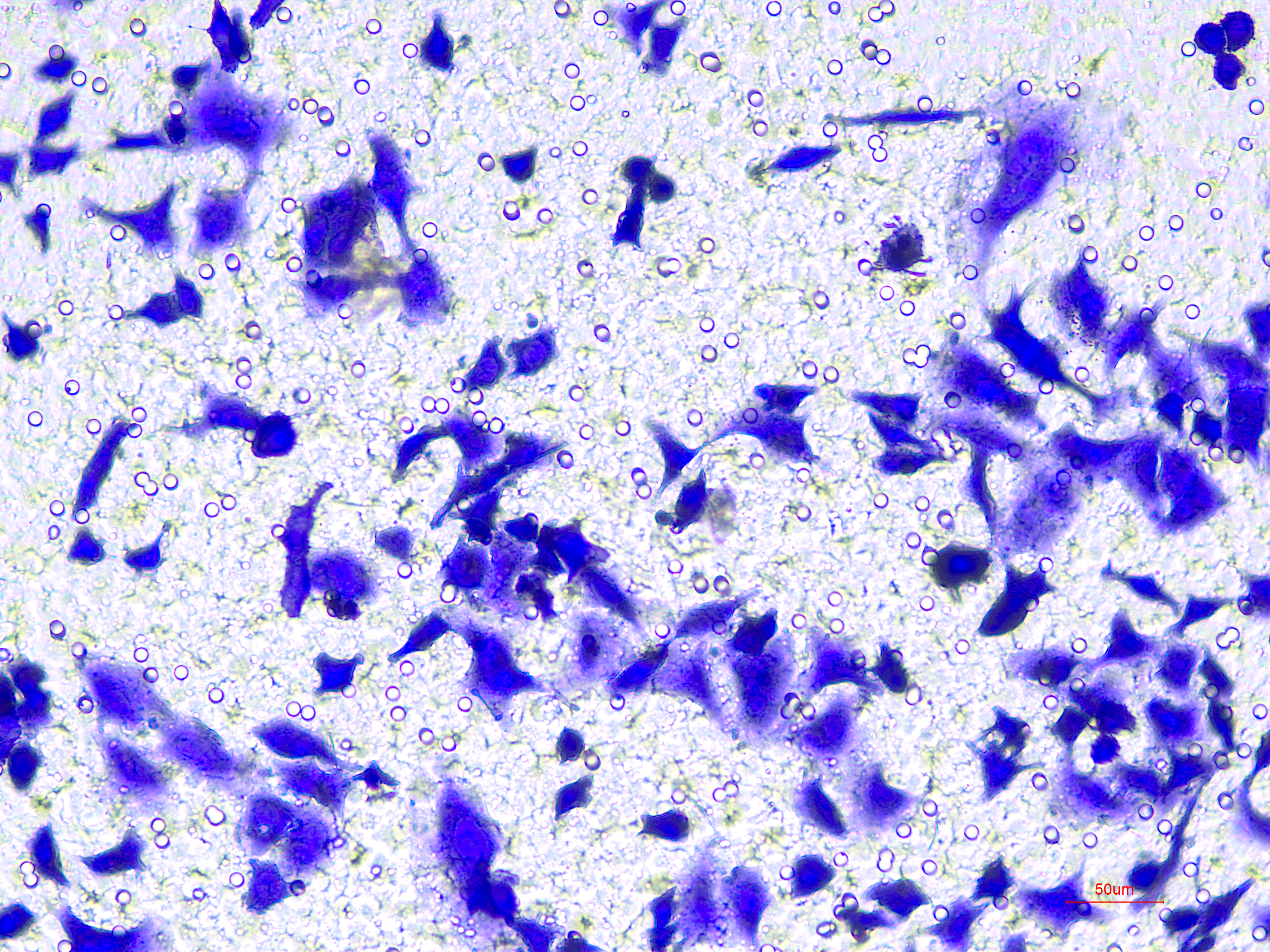
**

**UM1 si-circGOLPH3 Invasion**

**
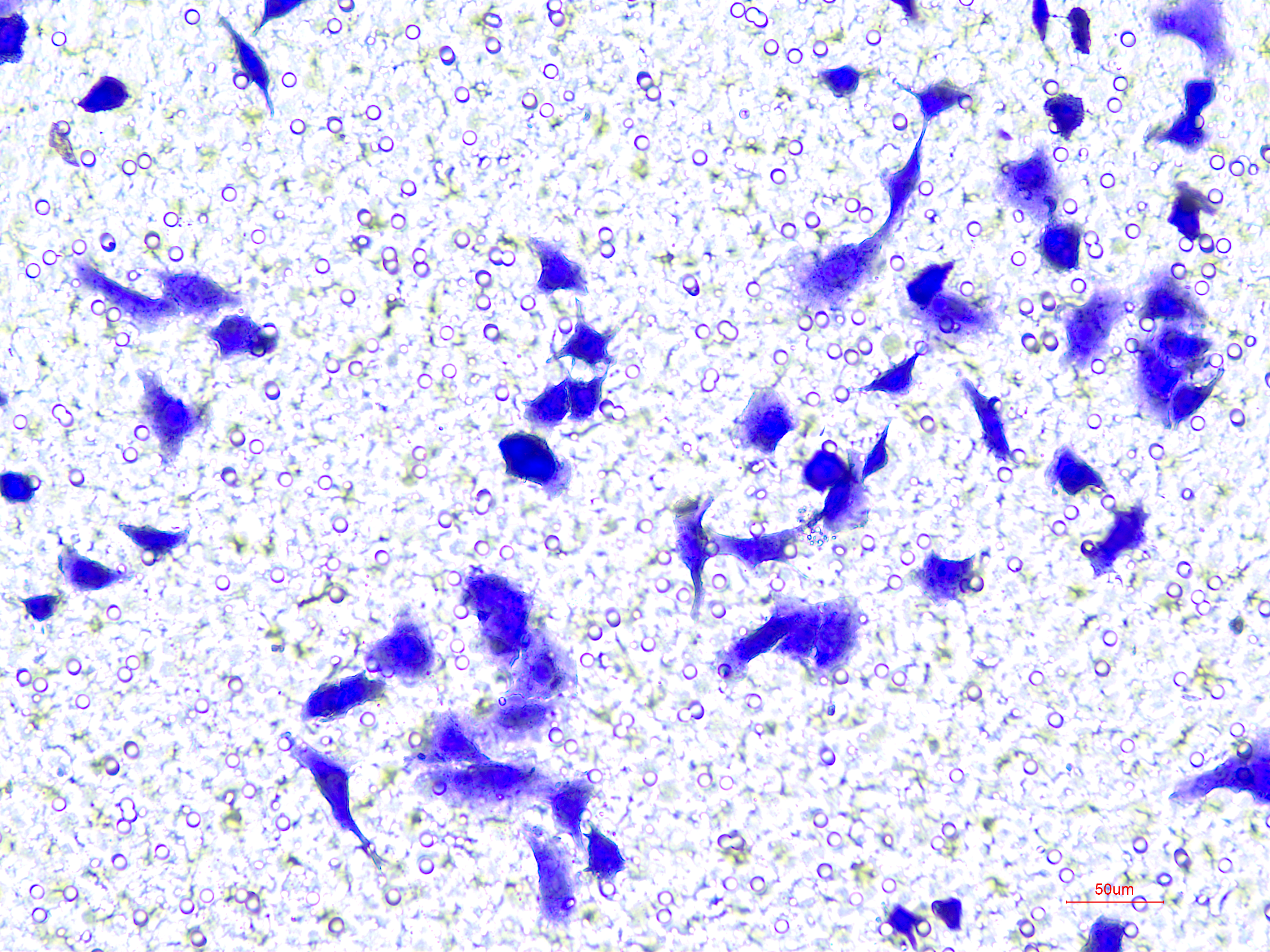
**

**UM1 si-cirGOLPH3+NC Invasion**

**
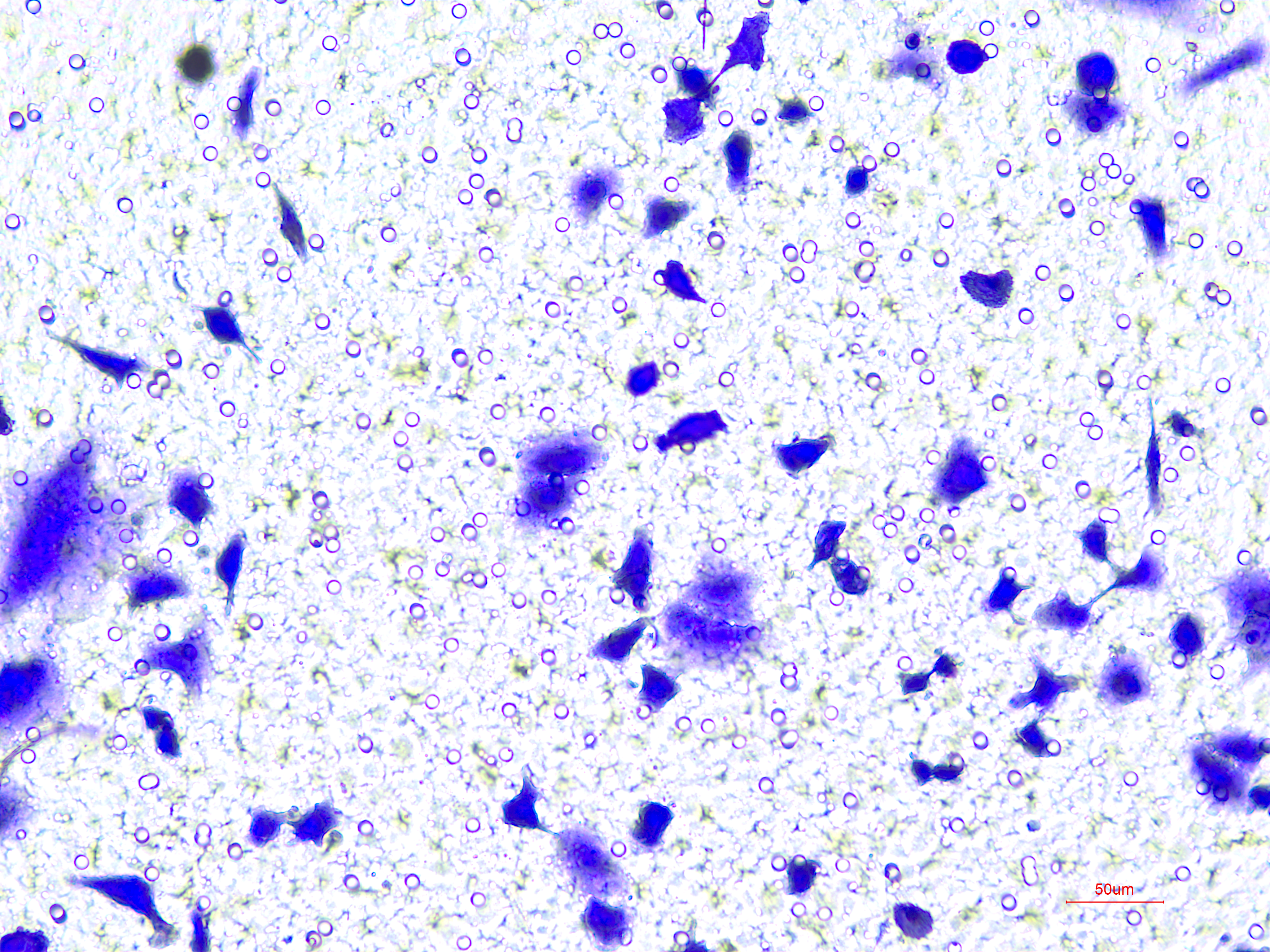
**

**UM1 si-circGOLPH3+miR-1299 Inhibitor invasion**

**
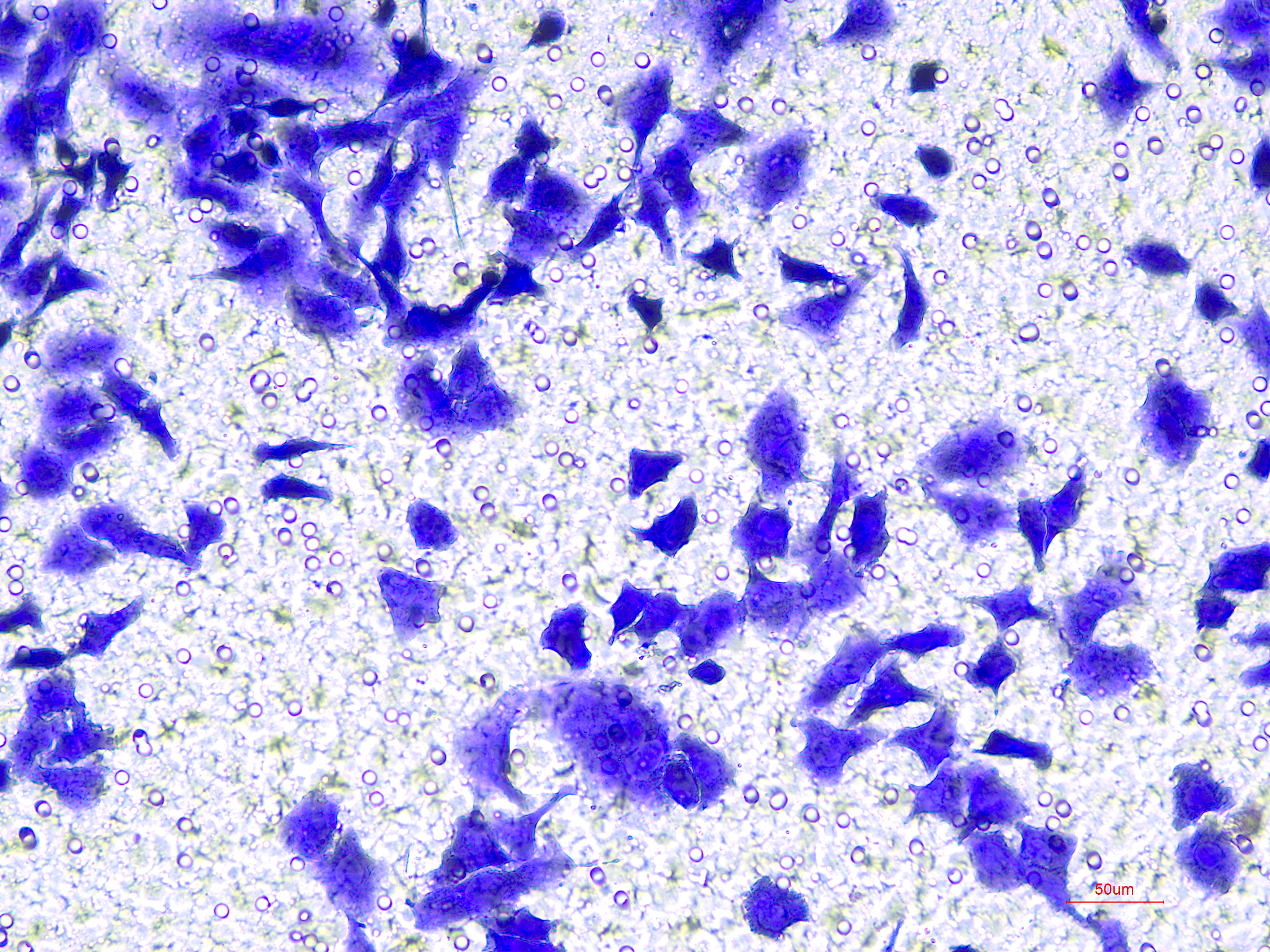
**

**UM1 si-circGOLPH3 +Vector invasion**

**
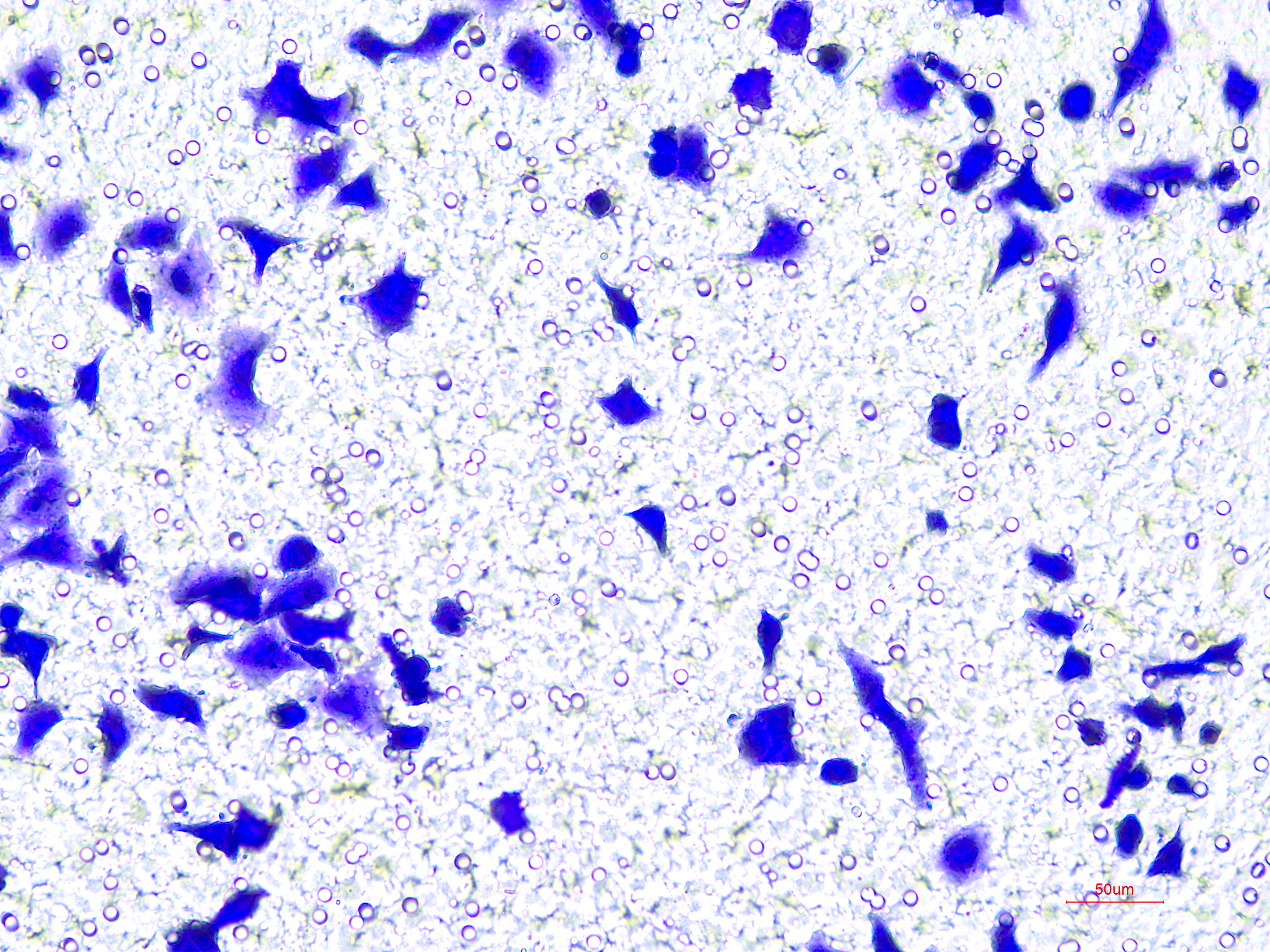
**

**UM1 si-circGOLPH3+OE-lif invasion**

**
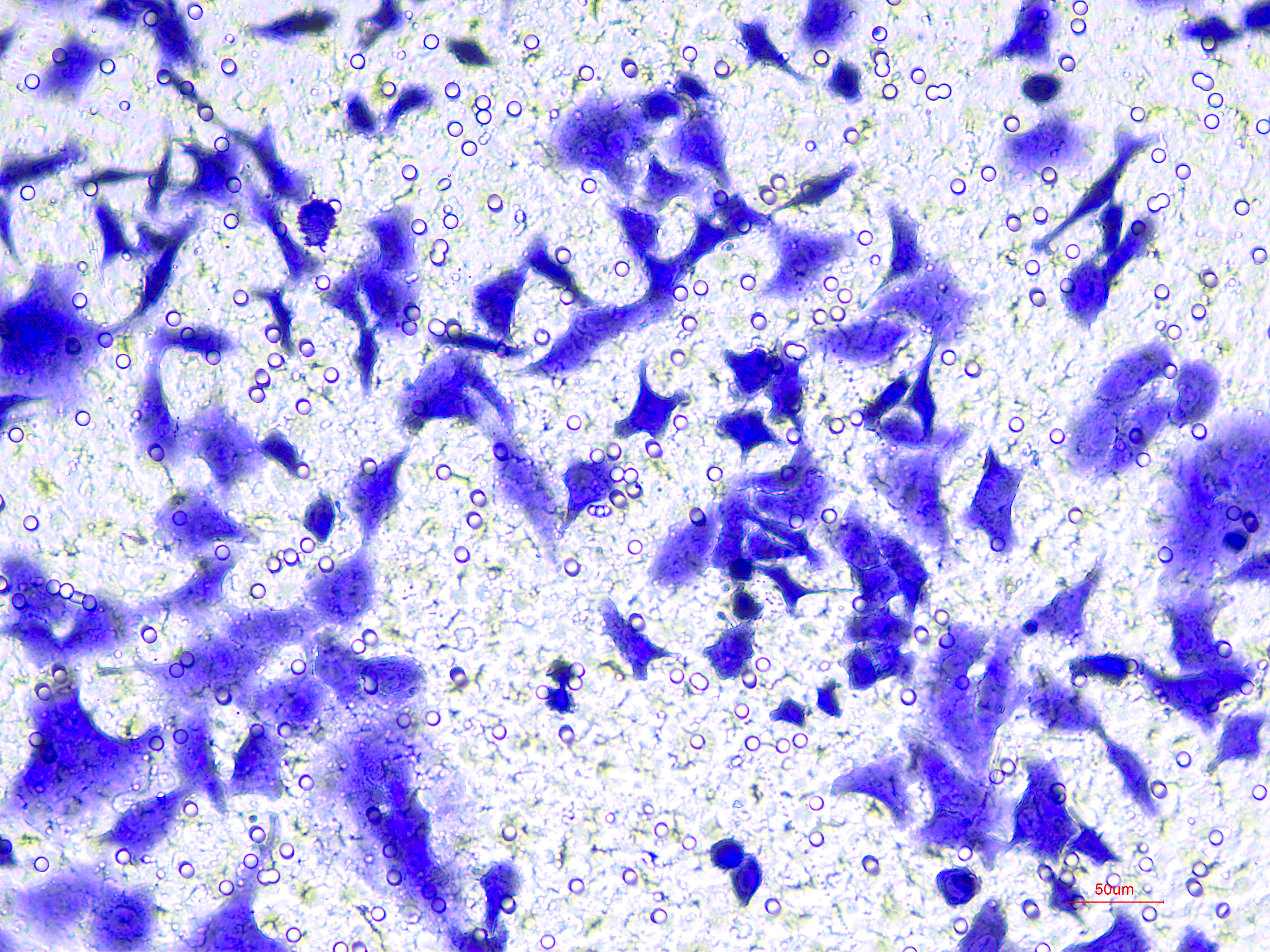
**

**Figure 5F**

**HSC3 Vector invasion**

**
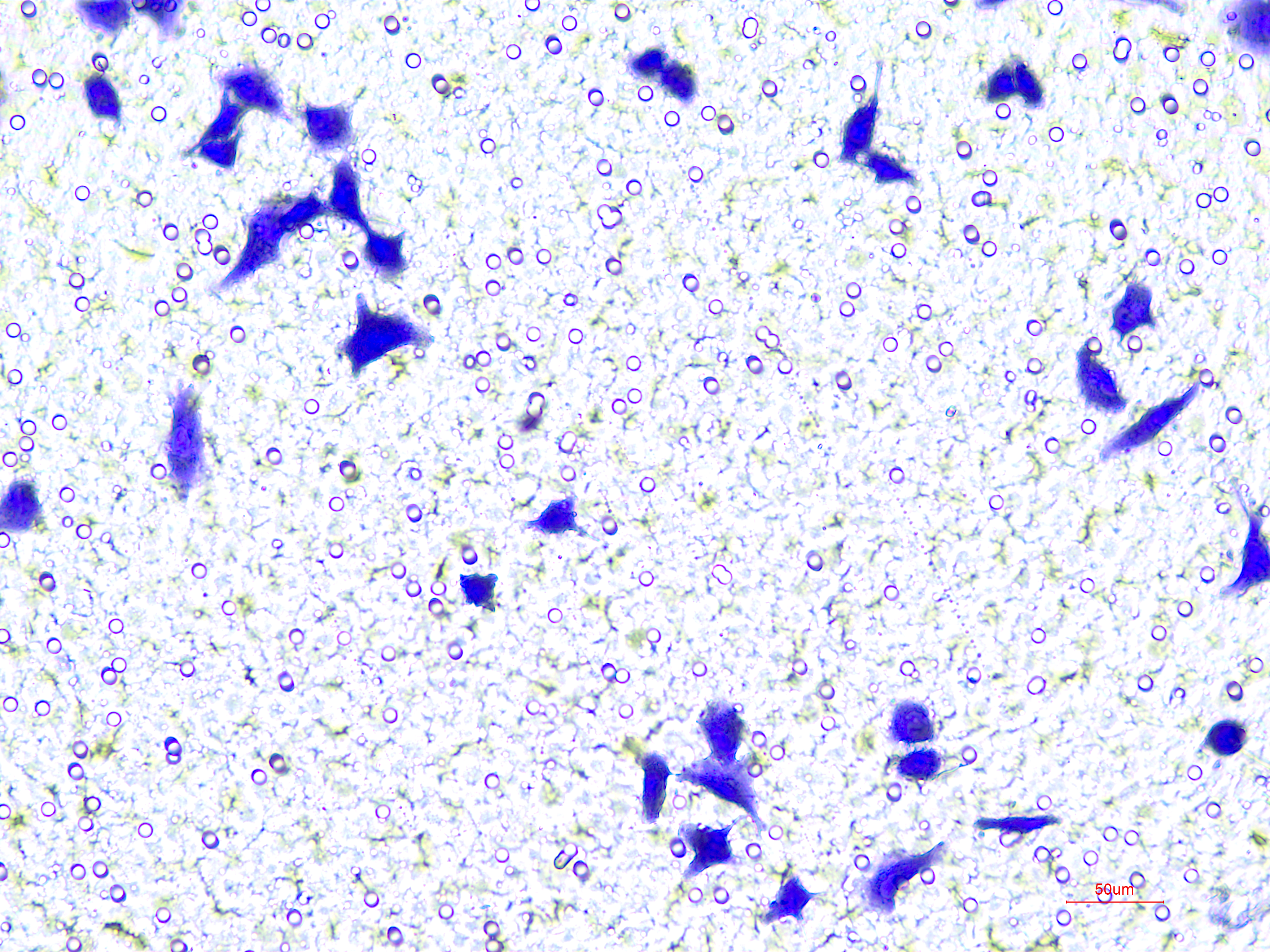
**

**HSC3 OE-circGOLPH3 Invasion**

**
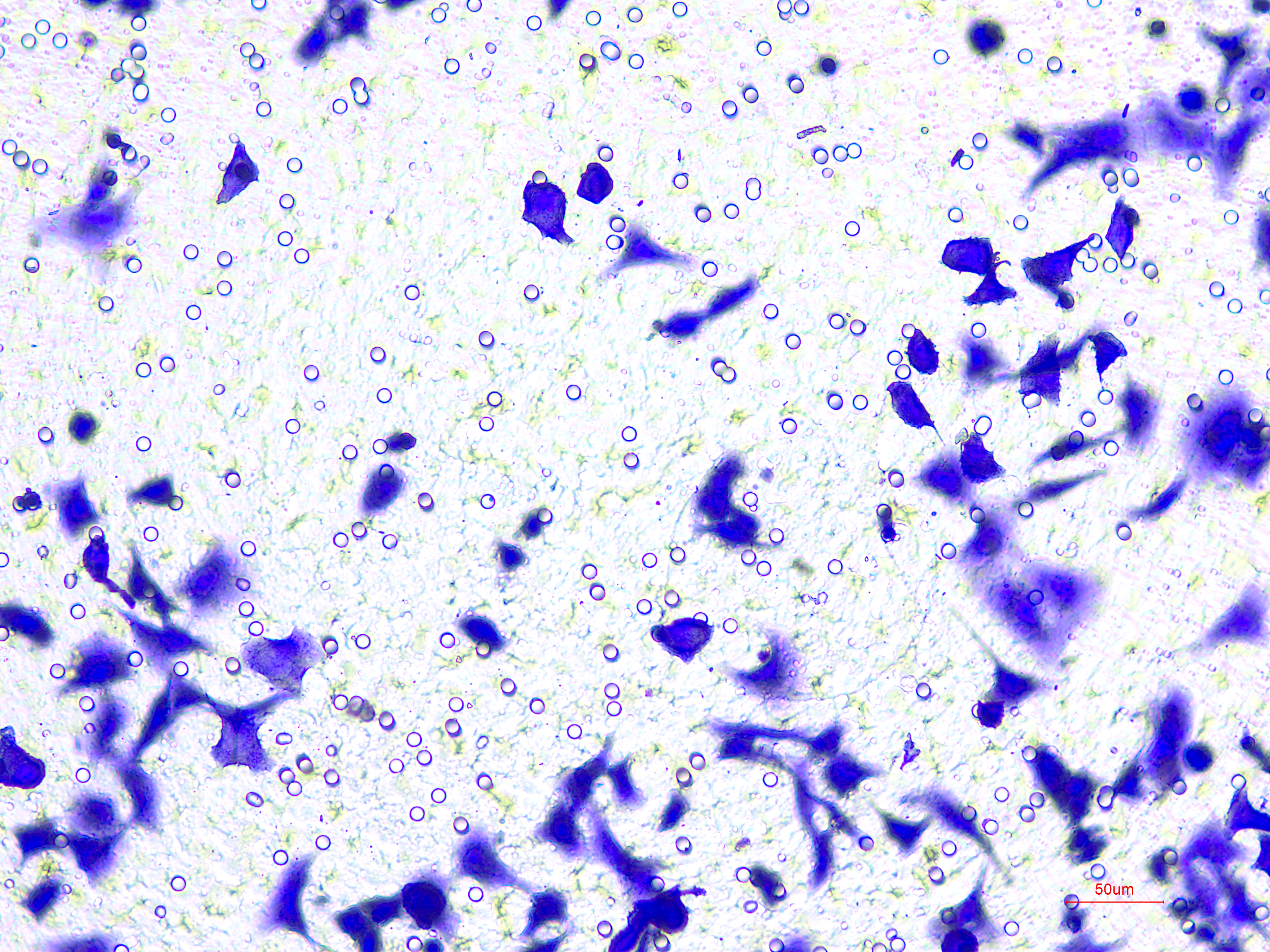
**

**HSC3 OE-circGOLPH3 +Vector Invasion**

**
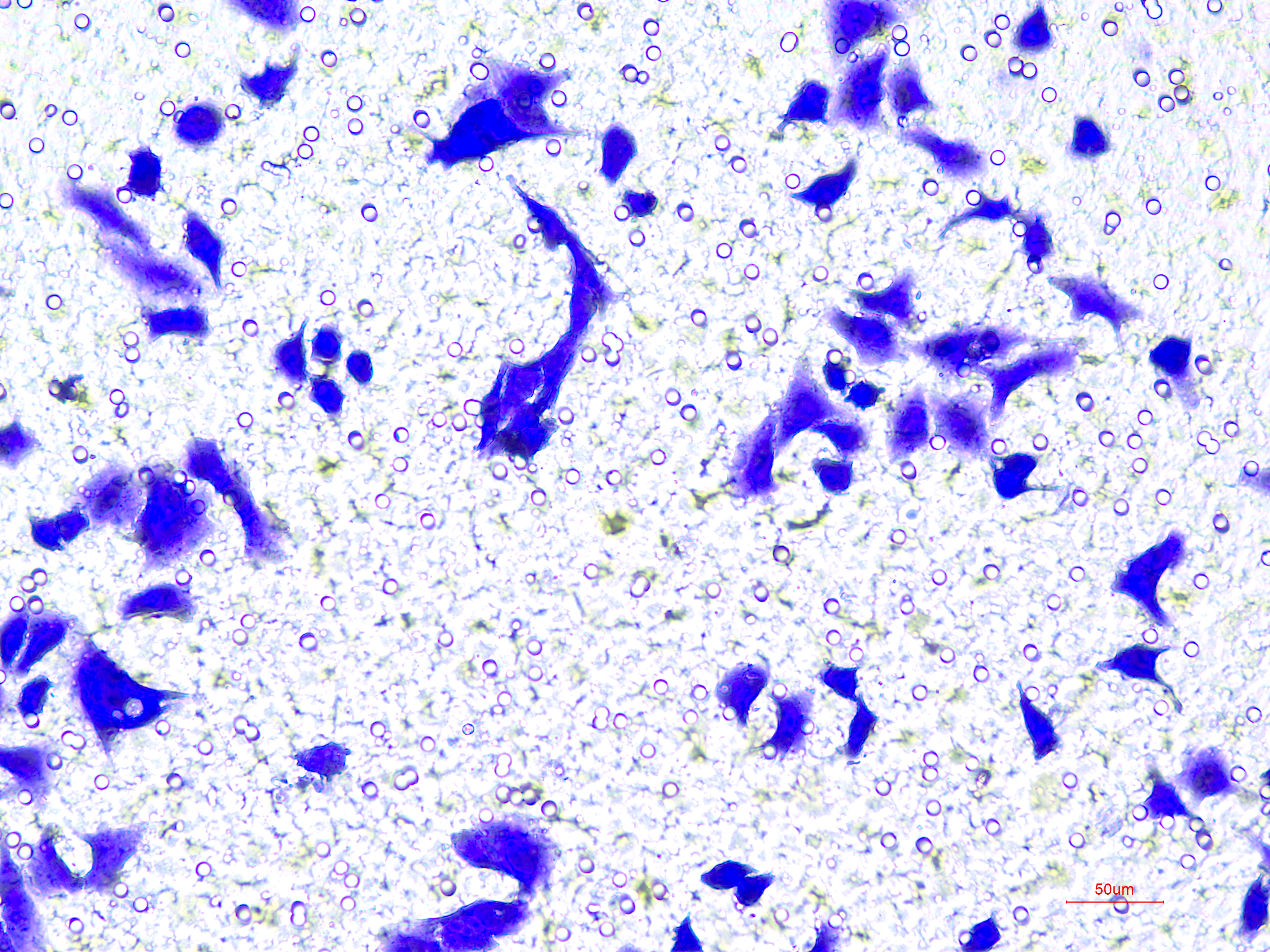
**

**HSC3 OE-circGOLPH3 +miR-1299 mimics invasion**

**
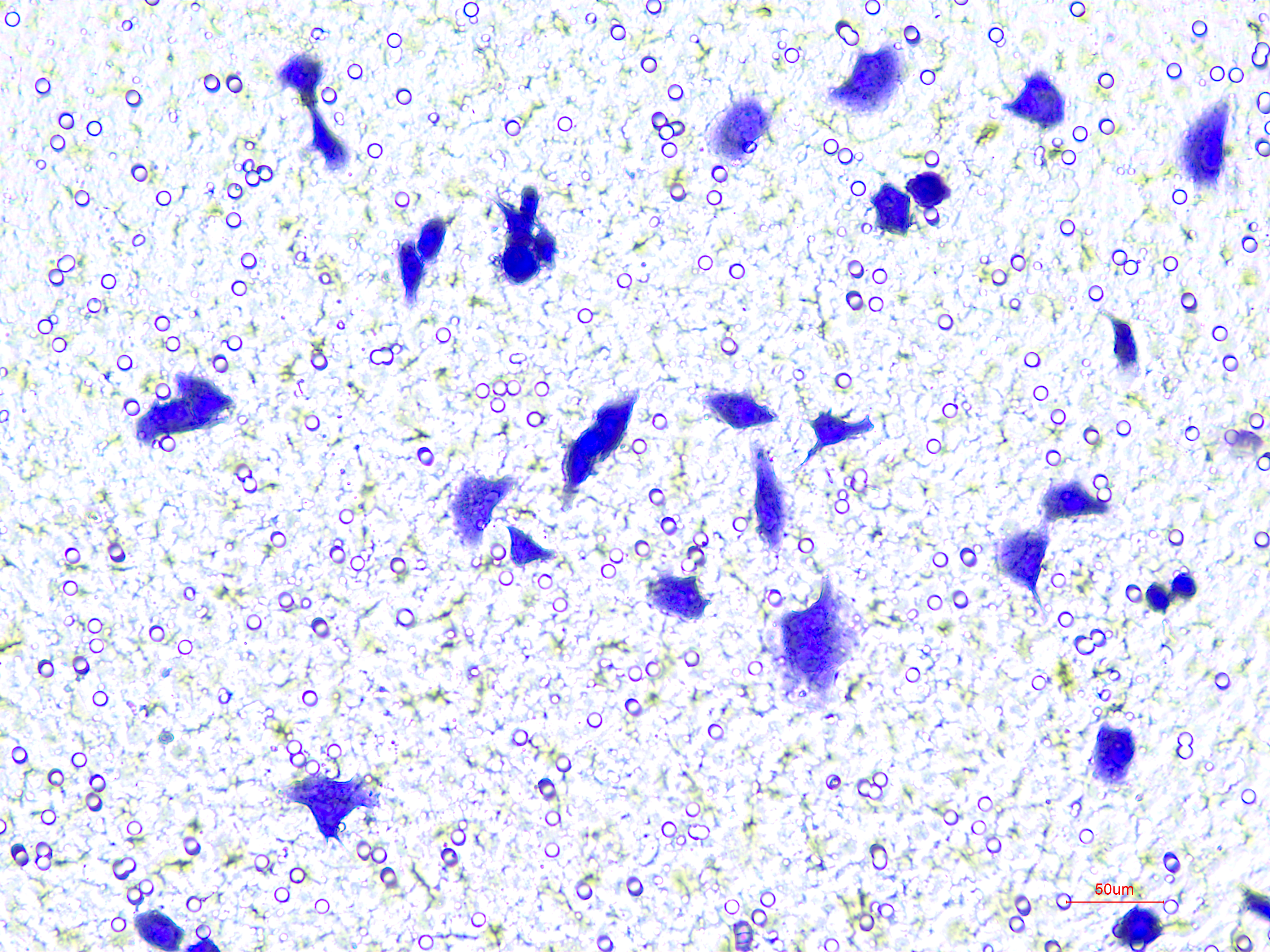
**

**HSC3 OE-circGOLPH3 +NC invasion**

**
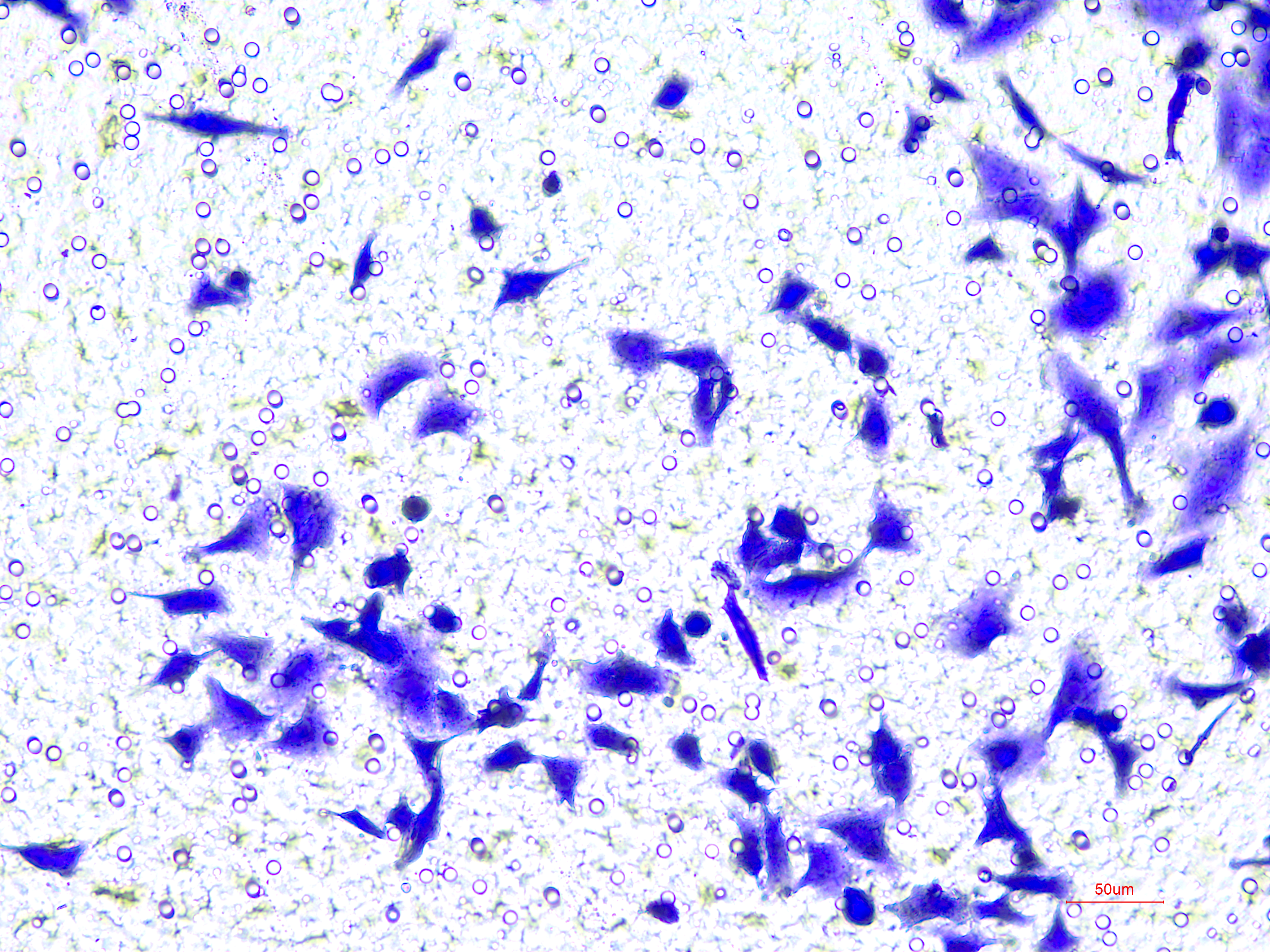
**

**HSC3 OE-circGOLPH3+SI-LIF Invasion**

**
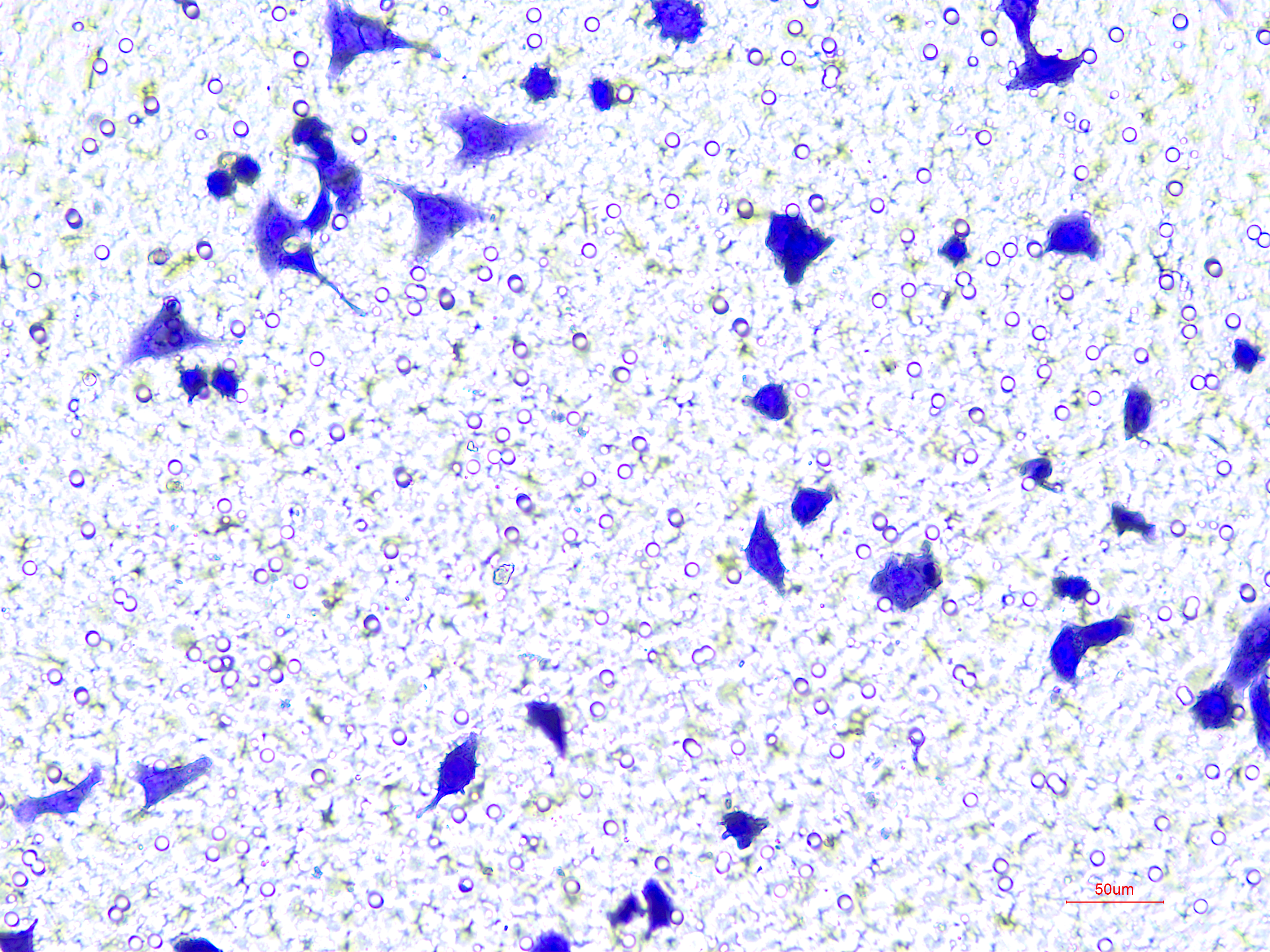
**
