# Supplementary figures and images for: Circular GOLPH3 RNA exerts oncogenic effects in vitro by regulating the miRNA-1299/LIF axis in oral squamous cell carcinoma
Source: Bioengineered. 2022 Apr 28;13(4):11012–25. doi: 10.1080/21655979.2022.2067288 (PMC9208457; doi:10.1080/21655979.2022.2067288)

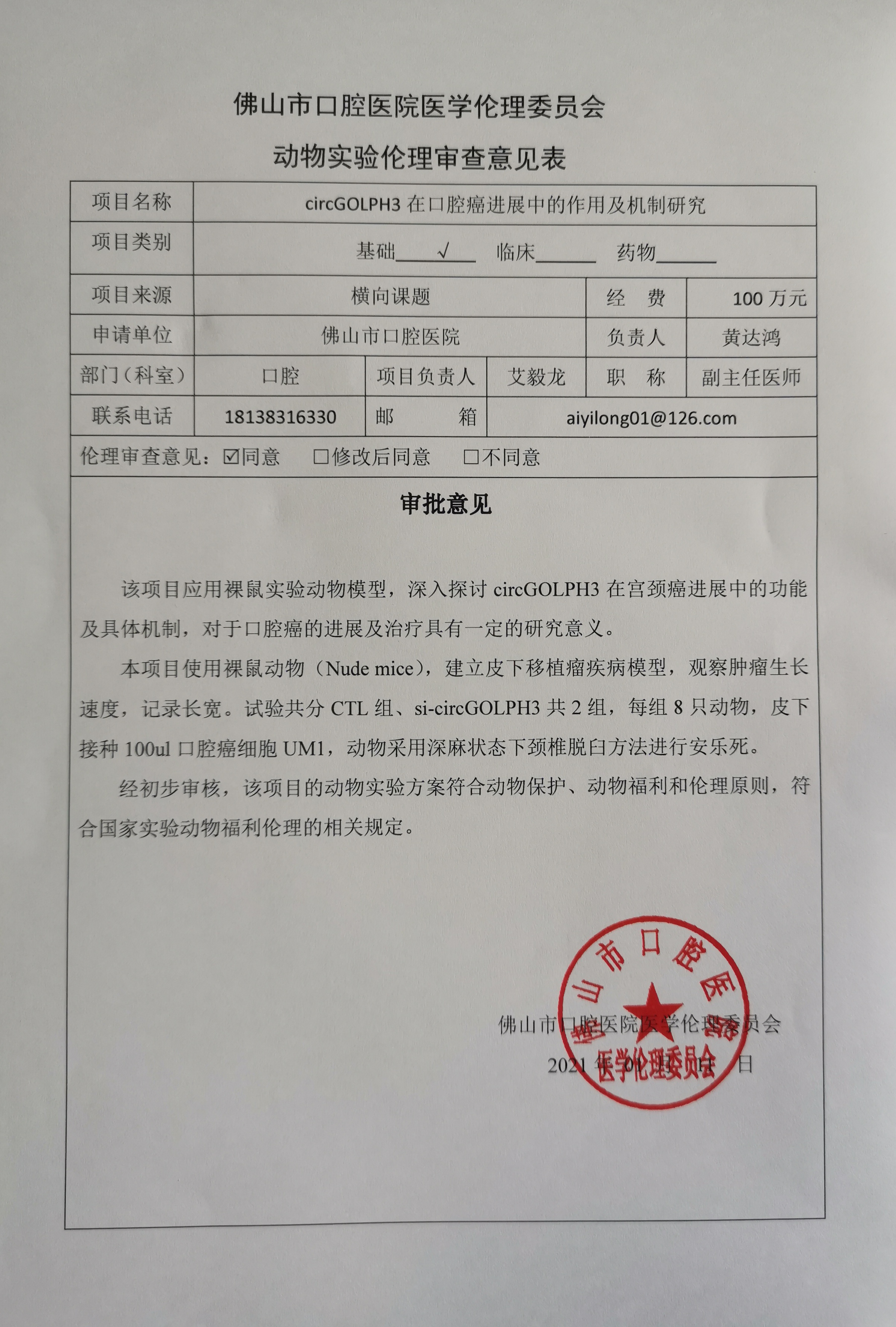

Supplement: Supplemental Material [file KBIE_A_2067288_SM7788.zip › supplementary/Ethical approvement.jpg]
